# Salivary dysbiosis in Sjögren’s syndrome and a commensal-mediated immunomodulatory effect of salivary gland epithelial cells

**DOI:** 10.1038/s41522-021-00192-w

**Published:** 2021-03-11

**Authors:** Yu-chao Tseng, Hsin-yi Yang, Wei-ting Lin, Chia-bin Chang, Hsiu-chuan Chien, Hon-pin Wang, Chun-ming Chen, Jann-tay Wang, Chin Li, Shu-fen Wu, Song-chou Hsieh

**Affiliations:** 1 Graduate Institute of Clinical Medicine, National Taiwan University, Taipei, Taiwan; 2 Division of Allergy, Immunology and Rheumatology, Department of Internal Medicine, Ditmanson Medical Foundation Chia-Yi Christian Hospital, Chiayi, Taiwan; 3 Center for Innovative Research on Aging Society (CIRAS), National Chung Cheng University, Chiayi, Taiwan; 4 Ditmanson Medical Foundation Chia-Yi Christian Hospital, Chiayi, Taiwan; 5 Department of Medical Research, Ditmanson Medical Foundation Chia-Yi Christian Hospital, Chiayi, Taiwan; 6 Department Oral and Maxillofacial Surgery, Ditmanson Medical Foundation Chia-Yi Christian Hospital, Chiayi, Taiwan; 7 Department of Urology, Ditmanson Medical Foundation Chiayi Christian Hospital, Chiayi, Taiwan; 8 Department of Laboratory Medicine, Ditmanson Medical Foundation Chia-Yi Christian Hospital, Chiayi, Taiwan; 9 Department of Internal Medicine, National Taiwan University Hospital, Taipei, Taiwan; 10 Department of Biomedical Sciences, Institute of Molecular Biology, and Institute of Biomedical Sciences, National Chung Cheng University, Chiayi, Taiwan

**Keywords:** Dentistry, Clinical microbiology, Microbiome

## Abstract

Salivary gland epithelial cells (SGECs) have been implicated in the pathogenesis of Sjögren’s syndrome due to aberrant antigen-presentation function. This study examined the hypothesis that oral dysbiosis modulates the antigen-presentation function of SGECs, which regulates CD4 T cell proliferation in primary Sjögren’s syndrome (pSS). Saliva samples from 8 pSS patients and 16 healthy subjects were analyzed for bacterial *16S* ribosomal DNA. As a result, 39 differentially abundant taxa were identified. Among them, the phylum *Proteobacteria* comprised 21 taxa, and this phylum was mostly enriched in the healthy controls. The proteobacterium *Haemophilus parainfluenzae* was enriched in the healthy controls, with the greatest effect size at the species level. Treatment of A253 cells in vitro with *H. parainfluenzae* upregulated PD-L1 expression, and *H. parainfluenzae*-pretreated A253 cells suppressed CD4 T cell proliferation. The suppression was partially reversed by PD-L1 blockade. Among low-grade xerostomia patients, salivary abundance of *H. parainfluenzae* decreased in pSS patients compared to that in non-pSS sicca patients. Our findings suggest that *H. parainfluenzae* may be an immunomodulatory commensal bacterium in pSS.

## Introduction

Sjögren’s syndrome, a prevalent systemic autoimmune disease without known effective treatment, is characterized by “autoimmune epithelitis”. Salivary gland epithelial cells (SGECs), as structural components of the epithelium, actively participate in these autoimmune inflammatory processes rather than being bystanders^1–6^. The SGECs are implicated in the recruitment, activation, expansion, differentiation, survival, and maintenance of immune cells, formation of the ectopic germinal center, and serve as sources of autoantigens^3,4,6^. Several studies have provided evidences indicating the intrinsic activation of SGECs in parallel with inflammatory infiltrates^3^. Therefore, being able to orchestrate both innate and adaptive immune responses, SGECs are considered the hub of autoimmune inflammatory processes in Sjögren’s syndrome.

Modulation of immune response by SGECs is of particular interest as demonstrated by the aberrantly expressed class II MHC molecules^7–15^ and co-stimulatory molecules^15–19^. SGECs also induce proliferation of anti-CD3-stimulated CD4 T cells in vitro^17,20^. CD4 T cells are the major components of salivary gland mononuclear infiltrates in Sjögren’s syndrome^21^. Upon activation, CD4 T cells polarize into different lineages, including Th1, Th17, and follicular helper T cells. These polarizations are implicated in the major immunological features of Sjögren’s syndrome, such as IFN-γ-induced SGECs activation and dysfunction, B cell activation and differentiation, and ectopic germinal center formation^22,23^, and therefore, contribute to the development and progression of the disease^24^.

In recent years, studies based on saliva, oral washing, and buccal and tongue mucosa have suggested the involvement of oral dysbiosis in Sjögren’s syndrome^25–32^, with some researchers further suggesting it may have an active role in the pathogenesis of Sjögren’s syndrome. Given the proximity of the oral cavity to the SGECs microenvironment, the present study hypothesized that oral microbiota may modulate the antigen presentation of SGECs to regulate CD4 T cell function. In this study, the salivary microbiota of primary Sjögren’s syndrome (pSS) patients were characterized by analyzing the *16S* ribosomal DNA. Differential abundances were determined by linear discriminant analysis (LDA) effect size (LEfSe). A253 cells were treated with the selected bacteria, and the surface expressions of MHC molecules and co-receptors were determined using flow cytometry. Proliferation of CD4 T cells following coculture with bacteria-pretreated A253 cells was determined using carboxyfluorescein succinimidyl ester (CFSE) staining. Our findings indicated a possible role of *H. parainfluenzae* in the modulation of the antigen-presentation function of SGECs that in turn regulates CD4 T cell activation in pSS.

## Results

### Patient characteristics

The baseline characteristics of pSS patients and healthy controls are presented in Table 1. The median age for pSS patients was 58 years, as reported in previous studies^33^. All participants were women and nonsmokers. None of the participants had any history of antibiotics, immunomodulators/immunosuppressants, or corticosteroids use in the 3 months prior to saliva collection.

**Table 1:** Baseline characteristics of the study participants.

	pSS patients	Healthy controls
Number of cases	8	16
Age (year, IQR)	58 (48–65)	59 (45–68)
Women (*n*, %)	8 (100)	16 (100)
Smoking (*n*, %)	0 (0)	0 (0)
Autoimmune diseases other than pSS (*n*, %)	0 (0)	0 (0)
Received immunomodulators/immunosuppressants or corticosteroids in last 3 months (*n*, %)	0 (0)	0 (0)
Received antibiotics in last 3 months (*n*, %)	0 (0)	0 (0)
Duration of clinically apparent xerostomia (months, IQR)	12 (11–36)	0 (0–0)
Sialoscintigraphy grades^a^		
<grade II (*n*, %)	3 (43)	NA
=grade II (*n*, %)	3 (43)	NA
>grade II (*n*, %)	1 (14)	NA
ESSDAI (point, IQR)	0 (0–4)	NA
Serology tests		
ANA titer ≥ 1:160 (*n*, %)	2 (25)	NA
Positivity of anti-Ro (*n*, %)	4 (50)	NA
Positivity of anti-La (*n*, %)	2 (25)	NA
Positivity of RF (*n*, %)	2 (25)	NA
LSG biopsy focus score ≥1 (*n*, %)	4 (50)	NA

*IQR* interquartile range, *NA* not applicable, *pSS* primary Sjögren’s syndrome, *ESSDAI* European League Against Rheumatism Sjögren’s syndrome disease activity index, *LSG* labial salivary gland, *ANA* antinuclear antibody, *RF* rheumatoid factor.

^a^One patient fulfilled the classification criteria of pSS without a sialoscintigraphy.

The median duration of clinically apparent xerostomia, a surrogate marker for disease duration, was 12 months for the pSS patients. The median European League Against Rheumatism Sjögren’s syndrome disease activity index was 0, indicating low prevalence of extra glandular involvement. Four (50%) of the pSS patients were positive for anti-Ro, while two (25%) patients were positive for both anti-Ro and anti-La. The remaining four (50%) patients had a focus score ≥1 in their labial salivary gland biopsies. Since patients with recent use of immunomodulators/immunosuppressants or corticosteroids were not enrolled in the present study, shorter disease duration and milder disease presentation was expected in our patient cohort.

### Salivary microbiota diversity

The summarized statistics of alpha diversity at the genus and species levels are presented in Table 2. The Good’s coverage index was ~1 for every sample, irrespective of the group or taxonomic rank, indicating adequate sequencing depth. The microbiota richness did not show any differences between pSS patients and healthy controls, which continued to be insignificant when estimated with Chao1 and ACE.

**Table 2:** Alpha diversity at the genus and species levels.

Taxonomic rank	Genus							Species						
Indices	Good’s coverage	Richness	Chao1	ACE	Pielou	Shannon	Simpson	Good’s coverage	Richness	Chao1	ACE	Pielou	Shannon	Simpson
pSS patients														
Mean	1.00	72.75	89.97	91.94	0.47	2.87	0.76	1.00	202.13	255.40	259.57	0.60	4.58	0.92
SD	0.00	28.16	55.81	58.58	0.08	0.45	0.06	0.00	64.76	136.36	151.47	0.07	0.58	0.03
Healthy controls														
Mean	1.00	69.75	82.60	84.38	0.50	3.03	0.76	1.00	203.13	245.83	237.96	0.62	4.72	0.91
SD	0.00	16.02	24.10	25.04	0.08	0.51	0.08	0.00	43.54	65.07	61.56	0.06	0.52	0.05
*p* Value	0.874	0.741	0.652	0.658	0.419	0.451	0.987	0.989	0.964	0.816	0.621	0.575	0.546	0.756

*pSS* primary Sjögren’s syndrome.

**Fig. 1:**
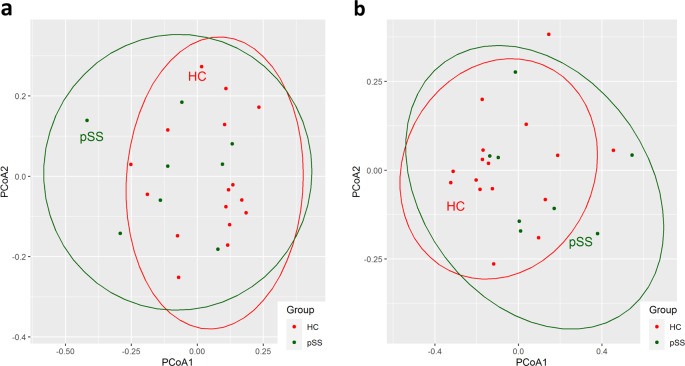
Principle coordinate analysis at the genus and species levels. Saliva samples from pSS patients (green) and healthy controls (red) could not be distinguished based on the microbiota at the **a** genus level and **b** species level. The distance matrix was computed using Euclidean distance.

The Pielou’s evenness index did not differ between groups, and the Shannon’s and Simpson’s diversity indices did not show statistically significant differences. Overall, the results for alpha diversity were similar to most oral microbiota studies regarding Sjögren’s syndrome^26,28–31^. Principle coordinate analysis using Euclidean distance showed that pSS patients could not be discriminated from healthy controls, either at genus or species level (Fig. 1).

### Identification of relevant taxa with differential abundance

The differential abundance for a specific taxon was analyzed using LEfSe, which provides not only *p* values, but also effect sizes represented as LDA scores. The low false discovery rate precludes the necessity for adjustment^34^. Differentially abundant taxa with LDA scores >2 were selected to ensure that only taxa having possible biological significance were reported. All the reported differentially abundant taxa had relative abundance >0.01% in at least one sample.

**Fig. 2:**
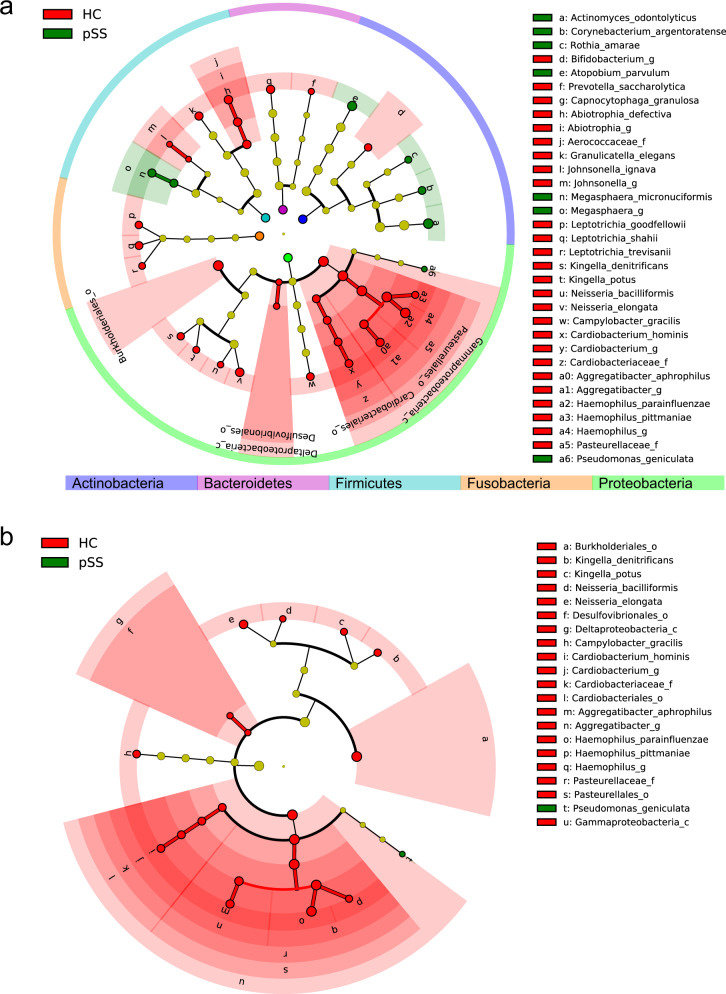
Cladogram of differentially abundant salivary microbiota between primary Sjögren’s syndrome patients and healthy controls by linear discriminant analysis effect size (LEfSe). Green nodes represent taxa enriched in pSS patients (“pSS”), while red nodes denote those enriched in healthy controls (“HC”). “p”, “c”, “o”, “f”, and “g” stand for phylum, class, order, family, and genus, respectively. From inside out, circles depict phylum, class, order, family, genus, and species, respectively. Only taxa of the final set (refer to Supplementary Table 1) or taxa of higher ranks related to the final set are presented **a** within all phyla and each phylum labeled with a differently colored ribbon, and **b** within the phylum Proteobacteria only.

Among the 1340 taxa detected, 61 taxa were differentially abundant between the pSS patients and the healthy controls. An adequate LDA score and a relevant relative abundance was ensured, following which a final set of 39 taxa, comprising 2 classes, 4 orders, 3 families, 7 genera, and 23 species were identified. The summarized filtering process and results are presented in Supplementary Fig. 1 and Supplementary Table 1. The taxonomic tree of the final set is presented in Fig. 2.

Of the 39 taxa identified, 33 were enriched in healthy controls, while six were enriched in pSS patients. These 39 taxa belonged to five different phyla, namely Actinobacteria, Bacteroidetes, Firmicutes, Fusobacteria, and Proteobacteria, but none of these phyla were differentially abundant between the two groups. Notably, out of the 39 taxa in the final set, Proteobacteria, comprised of 21 taxa (Fig. 2b), was mostly enriched in healthy controls, and accounted for almost two-thirds of the 33 taxa enriched in this group. In contrast, the only taxon belonging to Proteobacteria was enriched in pSS patients (Fig. 2b), accounting for only one-sixth of the six taxa enriched in this group. The tendency of Proteobacteria to be enriched mostly in healthy controls may indicate the shared biological properties of the taxa within this phylum. Further, this observation is in agreement with the decreased abundance of oral Proteobacteria reported for Sjögren’s syndrome patients^26,28,29,31,32^. Similarly, phylum Actinobacteria, comprised of four out of the six taxa belonging to the final set, were enriched in pSS patients.

### Differential abundance at the class, order, and family levels

The two classes identified in the final set, Gammaproteobacteria and Deltaproteobacteria, both belonged to phylum Proteobacteria and were enriched in healthy controls (Fig. 2b). Gammaproteobacteria, comprised of 13 taxa (12 taxa enriched in healthy controls and 1 taxon enriched in pSS patients), accounted for one-third of the taxa identified in the final set (Fig. 2b and Supplementary Table 1) and formed the largest cluster at the class level (Fig. 2). Although Deltaproteobacteria and Desulfovibrionales, belonging to class Gammaproteobacteria were represented by only two taxa in the final set, they were absent in pSS patients but were present in 8 out of the 16 healthy controls (*p* = 0.022, Fisher’s exact test), making them good candidates as biomarkers to exclude a diagnosis of pSS.

Pasteurellales and Cardiobacteriales, belonging to Gammaproteobacteria, and Burkholderiales, belonging to Betaproteobacteria (another class of Proteobacteria), were also enriched in healthy controls. Pasteurellales was represented by the family Pasteurellaceae, which comprised the two species of *Haemophilus* and one species of *Aggregatibacter* in the final set, forming a large subcluster (Fig. 2b). Firmicutes comprised eight taxa in the final set, accounting for more than one-fifth of all taxa. Moreover, six taxa were enriched in healthy controls and two taxa were enriched in pSS patients (Fig. 2a). Collectively, nine taxa from the class, order, and family levels were identified in the final set, indicating the presence of dysbiosis at higher taxonomic ranks.

### Differential abundance at the genus and species levels

The taxa at genus and species levels were focused upon to ensure precise validation of their biological significance. Since the effect size provides an estimation of the magnitude of the observed phenomenon, it is considered to be a valuable tool for ranking the relevance^34^. Thus, data are presented in the order of effect size, represented by the LDA scores in this study.

**Fig. 3:**
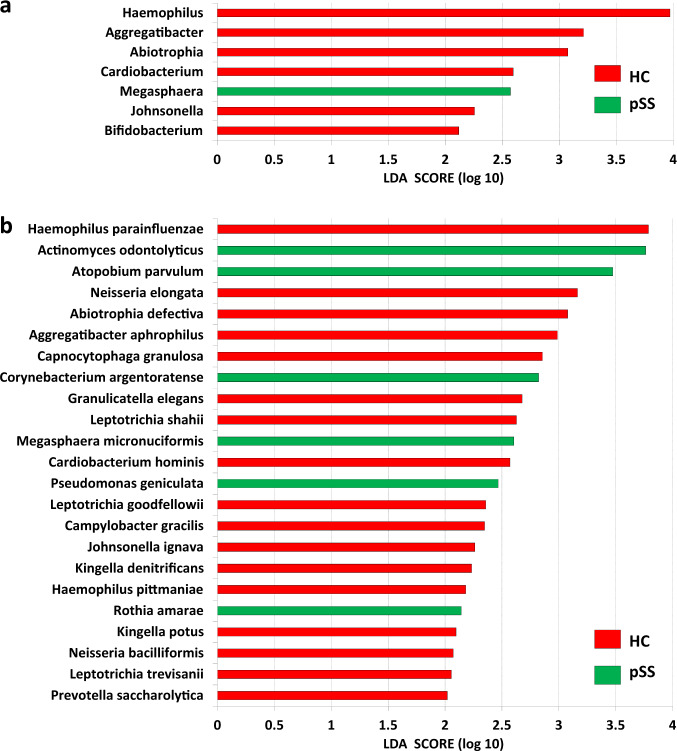
LDA scores of differentially abundant genera and species of salivary microbiota in primary Sjögren’s syndrome patients and healthy controls by linear discriminant analysis effect size (LEfSe). Green bars represent genera and species enriched in pSS patients (“pSS”), while red bars denote those enriched in healthy controls (“HC”). The genera and species are ordered by the LDA score, a measurement of effect size, at **a** the genus level and **b** the species level. *Haemophilus* and *H. parainfluenzae* had the highest LDA scores at the genus and species levels, respectively. Only genera and species of the final set (refer to Supplementary Table 1) are presented.

In general, 7 genera and 23 species were identified in the final set (Fig. 3a, b and Supplementary Table 1). *Haemophilus*, belonging to family Pasteurellaceae, had the greatest effect size (LDA score 3.97, enriched in the healthy controls, *p* = 0.012) at the genus level, and *H. parainfluenzae*, the major oral species within this genus had the greatest effect size (LDA score 3.79, enriched in the healthy controls, *p* = 0.017) at the species level. Genus *Aggregatibacter*, which also belongs to family Pasteurellaceae, accounted for the second highest effect size (LDA score 3.21, enriched in the healthy controls, *p* = 0.007) at the genus level. Altogether, the tendencies of *Haemophilus* and *Aggregatibacter* being more abundant in the healthy controls, with considerable LDA scores indicated a possible common biological property of genera belonging to Pasteurellaceae. Within the *Haemophilus* and *Aggregatibacter* genus*, Haemophilus pittmaniae* and *Aggregatibacter aphrophilus* (formerly known as *Haemophilus aphrophilus*^35^) were also enriched in the healthy controls. *Haemophilus* and *Aggregatibacter* constituted the majority of the family Pasteurellaceae (Supplementary Fig. 3), while *H. parainfluenzae*, accounted for most of the abundance in *Haemophilus* (Supplementary Fig. 4), justifying the observed LDA scores of the Pasteurellaceae family (LDA score 4.04, enriched in the healthy controls, *p* = 0.014; Supplementary Table 1) and *Haemophilus* genus.

Genera *Abiotrophia*, *Cardiobacterium*, *Megasphaera*, and *Johnsonella*, belonging to phylum Firmicutes (Fig. 2a and Supplementary Table 1) presented the next four highest LDA scores. *Abiotrophia*, *Cardiobacterium*, and *Johnsonella* were enriched in the healthy controls, while *Megasphaera* was enriched in the pSS patients. The species identified in these genera were *Abiotrophia defectiva*, *Cardiobacterium hominis*, and *Johnsonella ignava*, and *Megasphaera micronuciformis*. *A. defectiva* was enriched in the healthy controls and occupied the fifth place at the species level. *Granulicatella elegans* was the other species within Firmicutes identified in the final set. Genus *Bifidobacterium* in phylum Actinobacteria was enriched in the healthy controls.

For other species identified in the final set (Fig. 3b and Supplementary Table 1), *Actinomyces odontolyticus*, belonging to phylum Actinobacteria, had a LDA score similar to *H. parainfluenzae* (LDA score 3.76, enriched in the pSS patients, *p* = 0.032 for *A. odontolyticus*; LDA score 3.79, enriched in the healthy controls, *p* = 0.017 for *H. parainfluenzae*). This accounted for the second place at the species level and the first place among those enriched in the pSS patients. *Atopobium parvulum*, also an Actinobacterium, occupied the third place at the species level (LDA score 3.47, enriched in the pSS patients, *p* = 0.032). The two other species within Actinobacteria identified in the final set were *Corynebacterium argentoratense* and *Rothia amarae*. All these four species belonging to Actinobacteria were enriched in the pSS patients in contrast to *Bifidobacterium* genus (Fig. 2a).

Apart from those within the families Pasteurellaceae and Cardiobacteriaceae, six additional species belonging to Proteobacteria were identified in the final set, namely *Neisseria elongata, Neisseria bacilliformis*, *Kingella potus*, *Kingella denitrificans*, *Campylobacter gracilis*, and *Pseudomonas geniculata* (Fig. 2b and Supplementary Table 1).

The first four species belong to the Neisseriaceae family, indicating some shared biological properties. However, neither *Neisseria* or *Kingella*, nor family Neisseriaceae were identified in the final set. *N. elongata* was enriched in the healthy controls and accounted for the fourth place at the species level (Fig. 3b and Supplementary Table 1). *P. geniculata* was the only species belonging to Proteobacteria that was enriched in the pSS patients (Fig. 2b and Supplementary Table 1), suggesting a unique biological profile. The complete absence of *K. potus* and *N. bacilliformis* in pSS patients was another valuable finding (no *K. potus* in the pSS patients but present in 8 out of the 16 healthy controls, *p* = 0.022, Fisher’s exact test; no *N. bacilliformis* in the pSS patients but present in 8 out of 16 healthy controls, *p* = 0.022, Fisher’s exact test). The combination of *K. potus* and *N. bacilliformis* with class Deltaproteobacteria or order Desulfovibrionales further added to the discriminative power, as none of these taxa were present in the pSS patients, but appeared in 13 out of the 16 healthy controls (*p* < 0.001, Fisher’s exact test), warranting further studies in biomarker development. The four species belonging to phyla Bacteroidetes and Fusobacteria (Fig. 2a and Supplementary Table 1) were all enriched in the healthy controls (Fig. 3b).

A summary of the results at the genus and species levels shows that most of the genera and species belonging to Proteobacteria were enriched in the healthy controls, among which *Haemophilus* and *H. parainfluenzae* had the greatest LDA scores at the genus and species levels, respectively. Species within phylum Actinobacteria, were enriched in the pSS patients, in which *A. odontolyticus* and *A. parvulum* occupied the second and third places, according to LDA scores at the species level. Species in phyla Firmicutes, Bacteroidetes, and Fusobacteria were generally enriched in the healthy controls, and had lower LDA scores.

### Selection of appropriate bacterial species

The magnitudes of the associations, the taxonomic patterns of associations, and results from external studies were reviewed to find relevant bacterial species for further studies. Since the LDA score, a measurement of effect size, represents the magnitude of association, the candidate species were narrowed down by limiting species to those with an LDA score >3, and our results identified *H. parainfluenzae* (LDA score 3.79, enriched in the healthy controls), *A. odontolyticus* (LDA score 3.76, enriched in the pSS patients), *A. parvulum* (LDA score 3.47, enriched in the pSS patients), *N. elongata* (LDA score 3.16, enriched in the healthy controls), and *A. defectiva* (LDA score 3.08, enriched in the healthy controls; Fig. 3b and Supplementary Table 1).

In the analysis of the taxonomic patterns of association, a species in the final set was regarded as more relevant and less likely to be identified just by chance if additional species within the same genus or family were also identified in the final set, a concept similar to overrepresentation in gene set analysis. The species with relative abundance >0.01% in at least one sample were the only ones included, similar to the process of identification of the final set, to reduce the effect of minor species. Briefly, at the genus level, one, zero, zero, one, and zero additional species were reported in the final set, for *H. parainfluenzae*, *A. odontolyticus*, *A. parvulum*, *N. elongata*, and *A. defectiva*, respectively (Supplementary Fig. 5A), while at the family level, two, zero, zero, three, and zero additional species were reported (Supplementary Fig. 5B). *A. parvulum* and *A. defectiva* at the genus level and *A. defectiva* at the family level were excluded from statistical analyses due to the low numbers of total species in the corresponding genera and families. Statistical differences were not found at the genus or family levels (Fisher’s exact test, *p* = 0.413 at the genus level, *p* = 0.331 at the family level).

As these five species showed no additional discriminative features, the study proceeded based on an extensive literature review, the details of which are provided in supplementary materials. Briefly, decreased oral *H. parainfluenzae* and *Haemophilus* abundances are extensively reported to be associated with autoimmune and chronic inflammatory diseases. In contrast, increase in oral *A. odontolyticus* and *A. parvulum*, and decrease in oral *N. elongata*, and *A. defectiva* and their related genera have been linked to autoimmune and chronic inflammatory diseases to a lesser extent. Therefore, *H. parainfluenzae* was further investigated for its ability to modulate antigen presentation in SGECs to regulate CD4 T cell activation in vitro.

### *Haemophilus parainfluenzae* induces PD-L1 expression of A253 cells

**Fig. 4:**
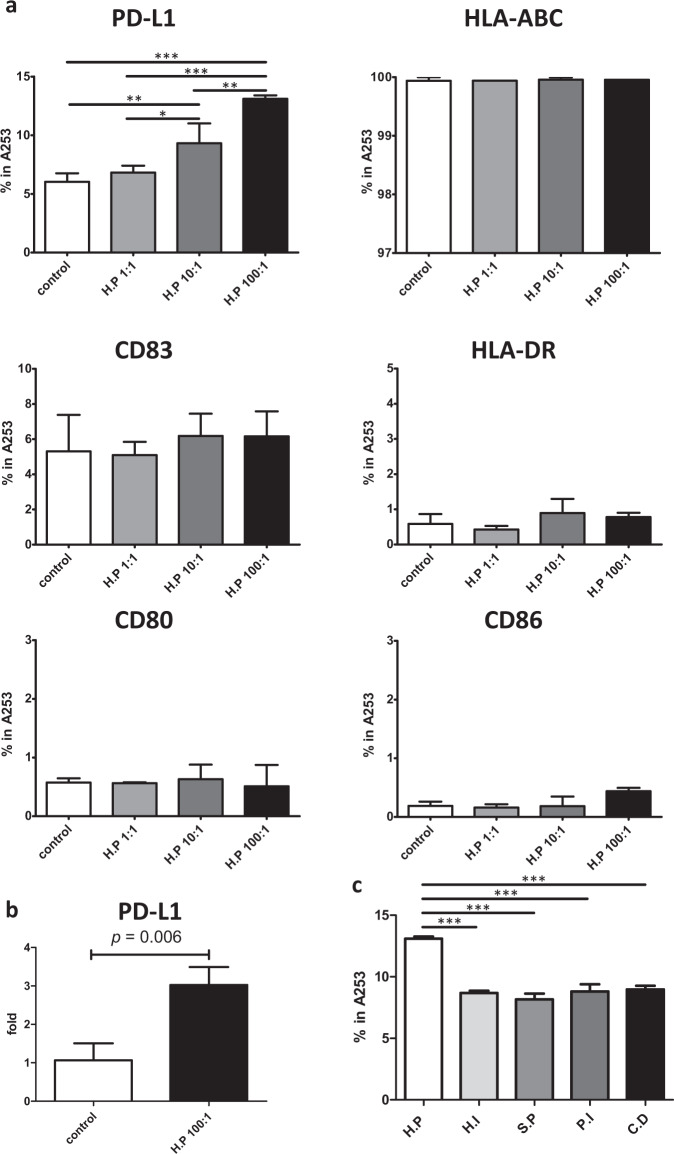
Increased expression of PD-L1 in *H. parainfluenzae*-treated A253 cells. **a** Increased percentage of cells expressing surface PD-L1 in a dose-dependent manner. One-way ANOVA *p* < 0.001; post hoc analysis, *p < 0.05, **p < 0.01, ***p < 0.001; percentages of cells with surface expression of HLA-ABC, CD83, HLA-DR, CD80, and CD86 were not changed. **b** Increased PD-L1 mRNA expression in *H. parainfluenzae*-treated A253 cells. *t* test *p* = 0.006. **c** Comparison of percentages of A253 cells expressing PD-L1 following treatment with various bacteria at a bacteria-to-cell ratio of 100:1. H.P *Haemophilus parainfluenzae*, H.I *Haemophilus influenzae*, S.P *Streptococcus pyogenes*, P.I *Prevotella intermedia*, C.D *Clostridium difficile*. One-way ANOVA, *p* < 0.001; post hoc analysis, ***p < 0.001; error bars stand for one standard error. Representative dot plots are provided in Supplementary Fig. 6.

The modulation of the antigen-presentation function of A253 cells by *H. parainfluenzae* was detected by examining the expressions of specific cell surface markers, following stimulation with heat-pretreated *H. parainfluenzae*. Among the markers tested, a differential expression of PD-L1 alone was detected (one-way analysis of variance (ANOVA), *p* < 0.001), while the other markers of antigen-presenting cell (APC) activation, including CD80, CD86, CD83, HLA-ABC, and HLA-DR, remained unchanged (Fig. 4a and Supplementary Fig. 6A). The percentage of cells expressing PD-L1 was consistently higher at the bacteria-to-cell ratio of 100:1 (mean ± SD: 6.0 ± 0.7%, 6.8 ± 0.6%, 9.3 ± 1.7%, and 13.1 ± 0.3% in control, 1:1, 10:1, and 100:1 respectively; post hoc analysis, *p* < 0.001 in control and 1:1 versus 100:1 and *p* < 0.01 in 10:1 versus 100:1). In addition, a dose-dependent trend was observed (Fig. 4a). Furthermore, PD-L1 mRNA expression was upregulated in A253 cells pretreated with *H. parainfluenzae* at the bacteria-to-cell ratio of 100:1 as compared to controls (mean ± SD: 3.02 ± 0.47 fold change at a bacteria-to-cell ratio of 100:1 and 1.06 ± 0.44 fold change in controls; *t* test, *p* = 0.006, Fig. 4b).

Additional bacteria were tested to determine whether the phenomenon of bacteria-induced PD-L1 upregulation on A253 cells was specific to *H. parainfluenzae*. Eight species, including two enriched in healthy controls and six enriched neither in pSS patients nor in healthy controls were selected. Bacteria enriched in the healthy controls, including *N. elongate* and *A. defectiva*, did not upregulate the surface PD-L1 expression in A253 cells (data not shown). Among bacteria not differentially abundant, *Haemophilus influenzae*, *Streptococcus pyogenes*, *Prevotella intermedia*, and *Clostridium difficile* significantly upregulated the surface PD-L1 expression, while *Staphylococcus aureus,* and *Veillonella parvula* did not (data not shown). Although bacteria-induced PD-L1 upregulation was not specific to *H. parainfluenzae*, A253 cells pretreated with *H. parainfluenzae* had the highest surface expression of PD-L1 among these bacteria (mean ± SD: 13.1 ± 0.3%, 8.7 ± 0.3%, 8.2 ± 0.8%, 8.8 ± 1.0%, and 9.0 ± 0.5% for *H. parainfluenzae*, *H. influenzae*, *S. pyogenes*, *P. intermedia*, and *C. difficile* respectively; one-way ANOVA, *p* < 0.001 and post hoc analysis, *p* < 0.001 in *H. parainfluenzae* versus all other bacteria; Fig. 4c and Supplementary Fig. 6B).

PD-ligand–PD-1 pathway activation contributes to the induction and maintenance of peripheral tolerance and protects against autoimmunity^36^. These results agreed with the clinical findings of decreased *H. parainfluenzae* abundance in pSS patients.

### *Haemophilus parainfluenzae-*pretreated A253 cells suppress CD4 T cell proliferation and is partially reversed by anti-PD-L1

**Fig. 5:**
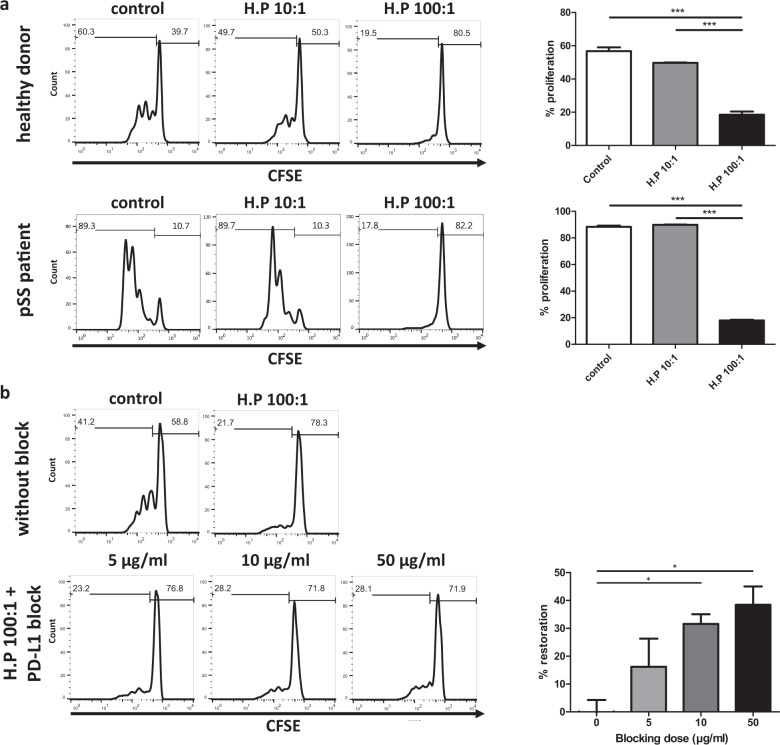
Inhibition of CD4 T cell proliferation by *H. parainfluenzae*-pretreated A253 cells and partial restoration by PD-L1 blockade. **a** CD4 T cell were coculture with *H. parainfluenzae*-pretreated A253 cells at various bacteria-to-cell ratios. CD4 T cells were isolated from peripheral blood mononuclear cells of healthy donors and anti-Ro-positive pSS patients. One-way ANOVA, *p* < 0.001; post hoc analysis, ***p < 0.001. **b** CD4 T cells isolated from healthy donors were cocultured with *H. parainfluenzae*-pretreated A253 cells at a bacteria-to-cell ratio of 100:1 and PD-L1 blocking antibody was added at various dosages. One-way ANOVA, *p* = 0.014; post hoc analysis, *p < 0.05. Error bars stand for one standard error.

As *H. parainfluenzae*-pretreated A253 cells showed robust increase of in PD-L1 expression, the antigen-presentation function was further evaluated by coculture experiment. To observe a presumed inhibitory effect, anti-CD3/28 beads were used to achieve full activation of CD4 T cells. A253 cells pretreated with *H. parainfluenzae* at the ratio of 100:1 of bacteria-to-cell, significantly suppressed the proliferation of CD4 T cells isolated either from healthy donors (mean ± SD: 56.8 ± 4.0%, 49.7 ± 0.5%, and 18.5 ± 3.3% in control, 10:1, and 100:1 respectively; one-way ANOVA, *p* < 0.001 and post hoc analysis, *p* < 0.001 in control and 10:1 versus 100:1; Fig. 5a), or from pSS patients (mean ± SD: 90.8 ± 4.0%, 87.7 ± 2.5%, and 12.5 ± 3.3% in control, 10:1, and 100:1 respectively; one-way ANOVA, *p* < 0.001 and post hoc analysis, *p* < 0.001 in control and 10:1 versus 100:1; Fig. 5a). In *H. parainfluenzae*-pretreated A253 cells, the blocking of PD-L1 at a dose of 10 and 50 μg/ml significantly restored CD4 T cell proliferation by almost 40% (mean ± SD: 0.0 ± 7.2%, 16.2 ± 17.5%, 31.6 ± 6.0%, and 39.5 ± 11.4% in no blocking, 5, 10, and 50 μg/ml respectively; one-way ANOVA, *p* = 0.014 and post hoc analysis, *p* < 0.05 in 10 and 50 μg/ml versus no blocking; Fig. 5b).

### Decreased salivary abundance of *H. parainfluenzae* in pSS patients compared to that in non-pSS sicca patients

Although a possible role of oral *H. parainfluenzae* in the contribution of altered antigen presentation was demonstrated, whether hyposalivation, a consequence of pSS, is sufficient to explain the association between decreased oral *H. parainfluenzae* and pSS, needed to be clarified. Because hyposalivation does not fulfill the classical definition of a confounding factor, a multivariate analysis with the inclusion of hyposalivation may alter the true association between the disease and the microbiota. Thus, another cohort consisting of low-grade xerostomia patients was studied.

Ten pSS patients and 11 non-pSS sicca patients were recruited (Supplementary Table 2). The distribution of age and sex was similar between the two groups. The median time of clinical apparent xerostomia did not exceed 12 months in both groups, indicating relatively shorter disease duration. All patients had sialoscintigraphy grades of less than or equal to grade II, the percentages of which were similar between the two groups. All non-pSS sicca patients tested negative for anti-Ro and anti-La and had focus scores <1 in their labial salivary gland biopsies.

The salivary microbiota of this cohort comprised 1809 taxa, of which 24 taxa were differentially abundant, following a similar filtering process. Detailed descriptions on the differentially abundant taxa are provided in Supplementary Table 3. Notably, salivary *H. parainfluenzae* remained to be less abundant in the pSS patients than in the non-pSS sicca patients (LDA score 4.00, *p* = 0.009). Therefore, factors other than hyposalivation were required to explain the association between decreased oral *H. parainfluenzae* and pSS. In other words, hyposalivation was not sufficient to explain this association.

## Discussion

The concept of immunomodulatory commensal bacteria has been proposed in recent years^37–40^. These bacteria, as permanent microbiota members, help to maintain and regulate the healthy immune steady state of the host^37^. The present study has provided some evidences for a role of oral *H. parainfluenzae* in maintaining immune homeostasis at the cellular level. The analysis of *16S* ribosomal DNA in saliva revealed a substantial decrease in the abundance of *H. parainfluenzae* in pSS patients. An induction of PD-L1 expression in A253 cells by *H. parainfluenzae* treatment, and a suppression of anti-CD3/28-induced CD4 T cell proliferation by *H. parainfluenzae*-pretreated A253 cells in vitro were also demonstrated. An extension of the study revealed that hyposalivation was not sufficient to explain the decreased salivary abundance of *H. parainfluenzae* in pSS patients. To the best of our knowledge, this is the first study to show the immunoregulatory role of *H. parainfluenzae*.

The oral commensal *H. parainfluenzae* was identified almost a century ago^41^, and it has been frequently regarded as an occasional pathogen. The present study evidenced a possible new role of *H. parainfluenzae* as an immunomodulatory commensal bacterium. Zhang et al.^40^ showed that the abundance of *Haemophilus* spp. (most-likely *H. parainfluenzae*) was negatively associated with the level of serum C-reactive protein, an inflammatory marker, in rheumatoid arthritis (RA) patients, which complements the findings of the present study. The serum titers of autoantibodies, such as anti-citrullinated antibody and rheumatoid factor, are negatively associated with some *Haemophilus* species in RA patients^40^ and with genus *Haemophilus* in high-risk individuals for RA^42^. Chen et al.^43^ also inferred that the abundance of *Haemophilus* species may protect children from Henoch-Schönlein purpura. Therefore, the present study provides additional evidence to support the role of *H. parainfluenzae* as an immunomodulatory commensal bacterium.

The present study also revealed that *H. parainfluenzae*-pretreated A253 cells suppressed CD4 T cell proliferation. Furthermore, *H. parainfluenzae* upregulated the expression of PD-L1 in A253 cells, while other markers responsible for APCs activation remained unchanged. The suppression of CD4 T cell proliferation was also partially reversed by PD-L1 blockade. Therefore, it is assumed that in a *H. parainfluenzae*-enriched environment, SGECs show an upregulated PD-L1 expression and suppress autoreactive CD4 T cells, thereby maintaining peripheral tolerance in terms of the autoimmune process.

Although aberrant expressions of class II MHC molecules and co-stimulatory molecules in SGECs were proposed to participate in the pathogenesis of pSS, the modulatory effect of SGECs has rarely been explored. Li et al.^44^ reported that SGECs isolated from non‐pSS sicca patients were able to suppress the proliferation of anti-CD3/28-stimulated CD4 T cells. The present study further added evidences by providing possible microbiota cues to the suppressive effect. Therefore, in addition to the enhanced APC function of SGECs, loss of the suppressive effect may also be important in the pathogenesis of pSS as well.

Recently, an oral microbiota study by Alam et al.^32^ showed that CD86 expression was downregulated in HSG cells by *Rothia mucilaginosa*, while IFN-γ-induced expressions of class II HLA, CD80, and CD86 were modulated by pretreatment with *Streptococcus salivarius*, *R. mucilaginosa*, *Fusobacterium nucleatum*, *Prevotella melaninogenica*, and *Prevotella histicola*. The present study, therefore, adds further evidence showing an upregulation of PD-L1 expression by *H. parainfluenzae* and several other bacteria, and suppression of CD4 T cell proliferation by *H. parainfluenzae*-pretreated A253 cells. A list of oral microbes capable of modulating SGECs may be reported as more focused, and detailed studies are performed in this field.

Despite the knowledge breakthroughs of the present study, it had several limitations. First, the effect of oral microbiota was only evaluated for some species, which did not exclude the effects of other components of microbiota. Many of the species enriched in the healthy controls are expected to have certain degrees of similarity. As most of them belong to Proteobacteria, they are phylogenetically related. Notably, more than half (9 out of 17) of the species enriched in the healthy controls, including four species from the *Haemophilus, Aggregatibacter, Cardiobacterium, Eikenella*, and *Kingella* (HACEK) group of microorganisms, are responsible for minor or rare causes of endocarditis^45–52^, while the six species enriched in the pSS patients have never been reported. This finding is not likely to be incidental, as several studies revealed similar negative associations of the HACEK bacteria in RA patients^40,53^. There may be some common critical features among these bacteria which await further exploration. The species *A. parvulum*, enriched in pSS patients, was found to have molecular mimicry with Ro60^54^ and has also been reported to induce pancolitis in colitis-susceptible interleukin-10-deficient mice^55^. Further studies focusing on the interaction of *A. parvulum* with SGECs are therefore worthwhile. Thus, despite smaller LDA scores of species other than *H. parainfluenzae*, it is still possible that any of these species or their combination may exhibit a modulatory effect on SGECs. Another limitation of this study was the small number of cases, which make it inadequate for the detection of subtle differences and prevent additional analyses. However, the impact of the data generated here asserts that this limitation did not severely compromise the study, since the primary objective of the study was to find relevant dysbiosis with the modulation effect on SGECs.

This study investigated the salivary dysbiosis in pSS patients and established the decrease of *H. parainfluenzae* as a major clinical feature in such patients. *H. parainfluenzae* upregulated the PD-L1 expression in A253 cells, and *H. parainfluenzae*-pretreated A253 cells suppressed CD4 T cell proliferation in vitro. Thus, *H. parainfluenzae* might be an immunomodulatory commensal in the pathogenesis of pSS. These findings provide significant insights into the possible protective roles of oral *H. parainfluenzae* in Sjögren’s syndrome, as well as in other autoimmune and chronic inflammatory diseases.

## Methods

### Study participants and saliva collection

Patients visiting the rheumatology clinic at the Ditmanson Medical Foundation Chia-Yi Christian Hospital for the evaluation of xerostomia, who fulfilled the 2002-revised American-European Consensus Group classification criteria for pSS^56^, were enrolled in the study. Healthy controls were recruited from the community. Individuals with a history of smoking, autoimmune diseases (except pSS in the case group), malignancies, diabetes mellitus, liver cirrhosis, and chronic kidney disease were excluded from the study.

Eight pSS patients and 16 healthy controls were enrolled in the study. Unstimulated whole saliva was collected from each participant at enrollment, as described previously^57^. All participants denied the use of antibiotics, mouth wash, corticosteroids, or medications for immune modulation or suppression within 3 months prior to saliva collection.

In the extension of the study, the cohorts consisted of pSS and non-pSS sicca patients limited to low-grade xerostomia. All non-pSS sicca patients were tested negative for anti-Ro and anti-La, and had focus scores <1 in their labial salivary gland biopsies. Both pSS and non-pSS sicca patients had sialoscintigraphy grades of less than or equal to grade II.

All human studies have been approved by the Research Ethical Committee of Ditmanson Medical Foundation Chia-Yi Christian Hospital (IRB106031), and all participants gave informed written consent for their enrollment in the studies. The work described has been carried out in accordance with the Declaration of Helsinki.

### DNA extraction and bacterial *16S* ribosomal DNA analysis

Saliva was resuspended in phosphate-buffered saline (PBS) and subjected to centrifugation. Undissolved debris were removed by low-speed centrifugation, and the saliva was washed twice in PBS before DNA extraction. The DNA of salivary microbiota was extracted with a QIAamp DNA Stool Mini Kit (Qiagen, Hilden, Germany), according to the manufacturer’s protocol. The concentration of purified DNA was determined by fluorometric spectrometry.

The protocol for *16S* ribosomal DNA sequencing given in the manufacturer’s (Illumina Inc., San Diego, CA, USA) manual was slightly modified for this study. Briefly, the variable regions 3 and 4 of the bacterial *16S* ribosomal DNA were amplified from the purified DNA specimens. Degenerate primers for annealing to the conserved bacterial *16S* ribosomal DNA sequences were adapted from a previous report. A set of mixed primers, with one to three nucleotides placed between their annealing and adaptor sequences, was used to increase the sequencing efficiency and data quality. The PCR products were separated by agarose gel electrophoresis and the expected-size products were gel-purified. A second-stage PCR using the Nextera XT index kit (Illumina Inc.) was performed to improve sequencing efficiency. Sequencing-ready libraries were analyzed by capillary electrophoresis and quantified by a fluorescence-based method. Sequencing was performed on the MiSeq platform (Illumina Inc.) for 18 dark and 350 read cycles for the forward read, and 18 dark and 250 read cycles for the reverse read.

The paired-end sequencing reads were trimmed using a quality score of Q20 as a threshold, and the forward and reverse reads were merged. Non-merged reads and merged reads shorter than 400 nucleotides were discarded. Trimmed and filtered reads were identified by using the basic local alignment search tool (BLAST) of the National Center for Biotechnology Information (NCBI) microbial *16S* database and the CLC Genomic Workbench v.8.5 (Qiagen Bioinformatics, Aarhus, Denmark). Only matching reads with at least 96% homology to the best-matched sequences were included in the subsequent analysis. The results were exported into R (https://www.r-project.org/) for further statistical analyses. Operational taxonomical units were identified using the Usearch package (https://www.drive5.com/usearch/).

### Preparation of A253 cells and treatment with bacteria

The A253 cells (ATCC HTB-41), derived from human submandibular glands with epithelial morphology and structure^58,59^, were cultured in McCoy’s 5A medium (ATCC) containing 10% fetal bovine serum (FBS). Similar to primary cultures of SGECs^16,18^, the A253 cells retain increased expression of HLA-ABC and HLA-DR following IFN-γ treatment (Supplementary Fig. 1).

*H. parainfluenzae*, acquired from the National Taiwan University Hospital (Taipei, Taiwan), was heat-treated for 2 h at 56 °C to inhibit the bacterial growth prior to incubation with A253 cells, as previously described^60^, and then washed and resuspended in Dulbecco’s PBS. A253 cells were then cocultured with heat-pretreated *H. parainfluenzae* at different bacteria-to-cell ratios for 24 h. *H. influenzae*, *S. pyogenes*, *S. aureus*, *C. difficile*, and *V. parvula* were acquired from the Department of Laboratory Medicine, Ditmanson Medical Foundation Chia-Yi Christian Hospital (Chiayi, Taiwan). *N. elongata* and *A. defectiva* were purchased from Leibniz Institute DSMZ-German Collection of Microorganisms and Cell Cultures.

### Measurement of A253 surface markers

The bacteria-treated A253 cells were incubated with fluorescent antibodies against PD-L1-allophycocyanin, CD80-phycoerythrin (PE), and CD86-PE (BD Biosciences, San Diego, CA, USA) and CD83-PE, HLA-ABC-Alexa488, and HLA-DR-Alexa488 (Biolegend, San Diego, CA, USA) for 30 min at 4 °C and analyzed by flow cytometry (BD FACSCaliber™, BD Biosciences, San Jose, CA, USA). Data were processed using FlowJo™ v10.6.2 (Becton, Dickinson & Company, Franklin Lakes, NJ, USA).

### Quantitative real-time PCR

Total RNA was extracted from *H. parainfluenzae*-treated A253 cells using Rezol™ C&T (Protech, Taipei, Taiwan), and cDNA was prepared using M-MLV reverse transcriptase with 1 μg of RNA. Primers for PD-L1 amplification were designed using Primer Expression v.3.0 (Applied Biosystems, Foster City, CA, USA). The FastStar Universal SYBR green Master mix (Roche Diagnostics GmbH, Mannheim, Germany), primers, and A253 cDNA were used for quantitative real-time PCR, which was performed on the ABI7500 instrument (Applied Biosystems).

### CD4 T cell proliferation assay and blocking of PD-L1

Bacteria-pretreated A253 cells were treated with 20 μg/ml mitomycin C for 45 min. CD4 T cells were isolated from the peripheral blood mononuclear cells obtained from healthy donors or anti-Ro-positive pSS patients by negative selection (BD IMag™ Human CD4 T Lymphocyte Enrichment Set). The isolated CD4 T cells were stained with 5 μM CFSE for 10 min and then washed twice with the T cell culture medium (RPMI-1640 with 1% l-glutamine, 1% penicillin–streptomycin, 10% FBS, 10 mM HEPES, and 50 μM β-mercaptoethanol). A253 cells and CD4 T cells were cocultured at a ratio of 1:5 with anti-CD3 and CD28 beads for 84 h. Suspended CD4 T cells were stained with 7-AAD (BD Biosciences, San Diego, CA, USA) and viable cells were analyzed for cell proliferation by flow cytometry.

For PD-L1 blocking, blocking antibody (Biolegend, San Diego, CA, USA) was added to the coculture. A reduction in the suppression of proliferation was normalized by the difference in proliferation between cocultures with bacteria-pretreated A253 cells at a bacteria-to-cell ratio of 100:1 and control A253 cells.

### Statistical analyses

All assays were performed using three technical replicates. Differential analysis of salivary microbiota was performed by LEfSe (http://huttenhower.sph.harvard.edu/galaxy/). Other statistical analyses were performed in SPSS for Windows v.21.0 (IBM Corp., Armonk, NY, USA). Comparisons of continuous data between groups were performed using Student’s *t* or Mann–Whitney *U* tests, and comparisons of categorical data were performed using Chi-squared or Fisher’s exact tests as appropriate. Multiple comparisons were evaluated by one-way ANOVA followed by Tukey’s or Kruskal–Wallis tests, as appropriate. Statistical significance was defined at *p* < 0.05.

### Reporting summary

Further information on research design is available in the Nature Research Reporting Summary linked to this article.

## Supplementary information

Supplementary Information

Reporting Summary

## Data Availability

The data files have been deposited in the NCBI Sequence Read Archive. The Bioproject accession numbers are PRJNA693659 and PRJNA693663.
