# Supplementary material for: Salivary dysbiosis in Sjögren’s syndrome and a commensal-mediated immunomodulatory effect of salivary gland epithelial cells
Source: NPJ Biofilms Microbiomes. 2021 Mar 11;7:21. doi: 10.1038/s41522-021-00192-w (PMC7952914; doi:10.1038/s41522-021-00192-w)
Supplement: Supplementary file 1 — Supplementary Information [file 41522_2021_192_MOESM1_ESM.pdf]

Supplementary Information for

Salivary dysbiosis in Sjögren's syndrome and a commensal-mediated modulation of salivary gland epithelial cells to inhibit CD4 T cell proliferation

Tseng et al.

List of contents

Supplementary Note.. 3

Supplementary Figures.. 5

Supplementary Figure 1.. 5

Supplementary Figure 2.. 6

Supplementary Figure 3.. 7

Supplementary Figure 4.. 8

Supplementary Figure 5.. 9

Supplementary Figure 6.. 10

Supplementary Figure 7.. 11

Supplementary Tables... 12

Supplementary Table 1... 12

Supplementary Table 2... 14

Supplementary Table 3... 15

Supplementary References 16

Supplementary Note

Literature review of candidate species

Five candidate species, namely *Haemophilus parainfluenzae* (LDA score 3.78, enriched in the healthy controls), *Actinomyces odontolyticus* (LDA score 3.76, enriched in the pSS patients), *Atopobium parvulum* (LDA score 3.47 enriched in the pSS patients), *Neisseria elongata* (LDA score 3.16, enriched in the healthy controls), and *Abiotrophia defectiva* (LDA score 3.08, enriched in the healthy controls) were reviewed extensively.

Haemophilus parainfluenzae, a fastidious gram-negative coccobacillus, frequently regarded as a minor cause of endocarditis[42,49], is a part of the normal human oral microbiota, accounting for most of the *Haemophilus* spp. in human saliva[61,62]. Surprisingly, the decrease in oral abundance of *H. parainfluenzae* has not only been linked to Sjögren's syndrome[32], but also to many of other autoimmune or chronic inflammatory diseases, such as systemic lupus erythematosus[63], rheumatoid arthritis (RA)[40,53], refractory celiac disease[64], autoimmune polyendocrine syndrome type-1[65], primary sclerosing cholangitis[66], Crohn's disease[67,68], and orofacial granulomatosis[68]. Decreased oral *Haemophilus* has also been reported in Sjögren's syndrome[26,31], RA[43], Henoch-Schönlein purpura[44], primary sclerosing cholangitis[66], ulcerative colitis[67], Crohn's disease[67,69,70], and palmoplantar pustulosis[71].

Actinomyces odontolyticus is a Gram-positive, facultative anaerobic bacterium initially isolated from dental caries[72]. Increased oral abundance of *A. odontolyticus* has been linked to RA[40] and psoriasis[73]. Some microbiota-based studies have also reported increased oral abundance of *Actinomyces* in anti-Ro+ mothers of neonatal lupus children[54], RA[43], type 1 diabetes[74], and IgA nephropathy[75].

Atopobium parvulum, originally *Peptostreptococcus parvulus*[76,77], is a non-motile and obligate anaerobic Gram-positive bacterium in the human oral cavity[78,79]. Oral *A. parvulum* has been more abundant in Sjögren's syndrome[28], anti-Ro+ mothers of

neonatal lupus children[54], RA[40,53], and Behçet disease[80]; it has also been more prevalent in Sjögren's syndrome[31]. Increased oral abundance of *Atopobium* has been observed among anti-Ro+ mothers of neonatal lupus children[54] and RA patients[43].

Neisseria elongata, an aerobic Gram-negative bacillus originally isolated from the human nasopharynx[81], has been reported as a rare cause of endocarditis[52]. Decreased oral *N. elongata* has been linked to Sjögren's syndrome[32], RA[40], and Crohn's disease or orofacial granulomatosis[68], whereas decreased oral abundance of *Neisseria* has been found in Sjögren's syndrome[26,28,31], RA[43], autoimmune polyendocrine syndrome type-1[65], IgA nephropathy[82], and Crohn's disease[67,70].

Abiotrophia defectiva, a facultatively anaerobic Gram-positive coccus[83], formerly known as *Streptococcus defectivus*[84], is a part of the normal flora of the human mouth[85], and again, a rare cause of endocarditis[51]. Studies have reported decreased oral abundance of *A. defectiva* in psoriasis[73] and *Abiotrophia* in Sjögren's syndrome[29].

In summary, decreased oral abundance of *H. parainfluenzae* and *Haemophilus* have been extensively reported in various autoimmune and chronic inflammatory diseases. This mini-review provided sufficient rationale for the prioritized investigation of *H. parainfluenzae*.

Supplementary Figures

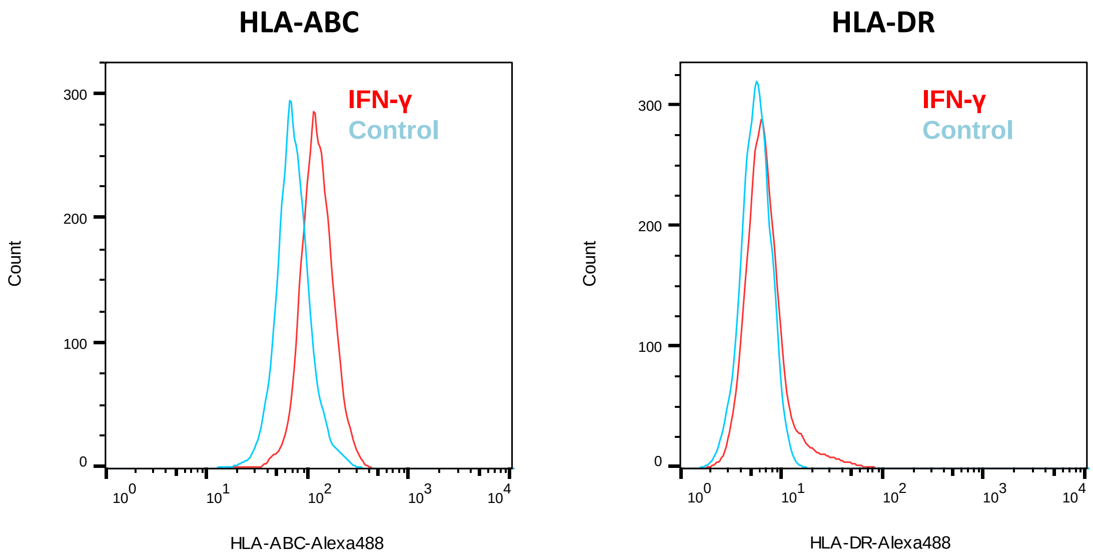

Supplementary Figure 1 Increased surface expression of HLA-ABC and HLA-DR on A253 cells after IFN-γ treatment. A253 cells were treated with IFN-γ at 10 IU/mL for 24 h. Representative histograms are shown.

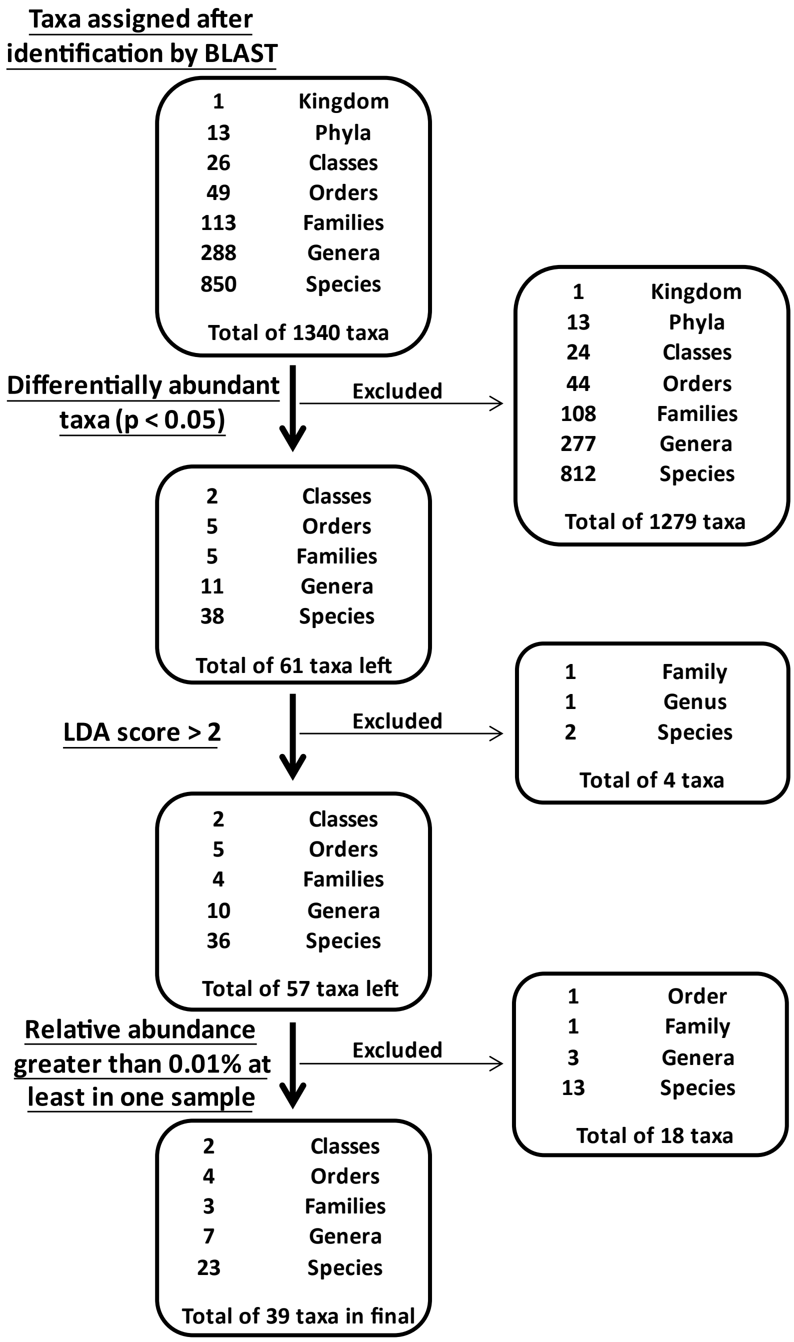

Supplementary Figure 2 The filtering process of relevant differentially abundant taxa in pSS patients and healthy controls. Starting with total identified 1340 taxa, 61 taxa were differentially abundant with LEfSe p values less than 0.05. Following filtering with LDA scores, 4 differentially abundant taxa were excluded for LDA scores ≤2. Of the 57 taxa left, 18 taxa did not meet the criteria of relative abundances greater than 0.01% at least in one sample. The final set consisted of 2 classes, 4 orders, 3 families, 7 genera, and 23 species, a total of 39 taxa.

a

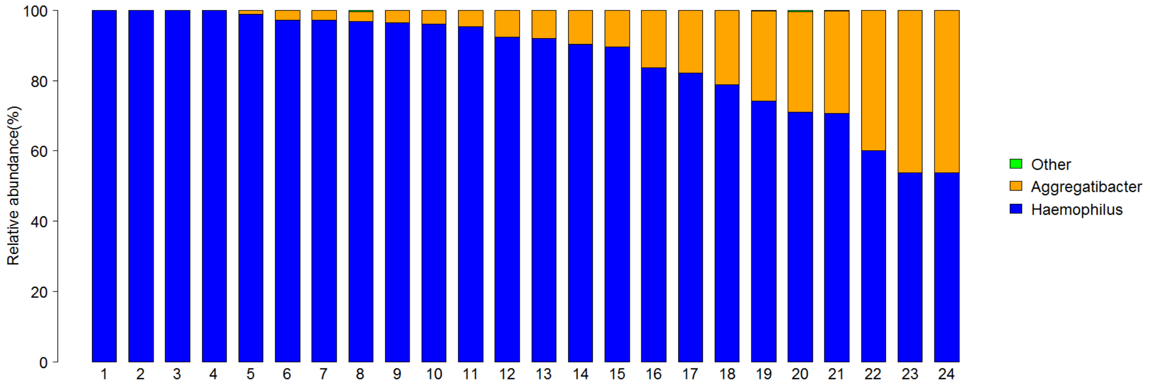

b

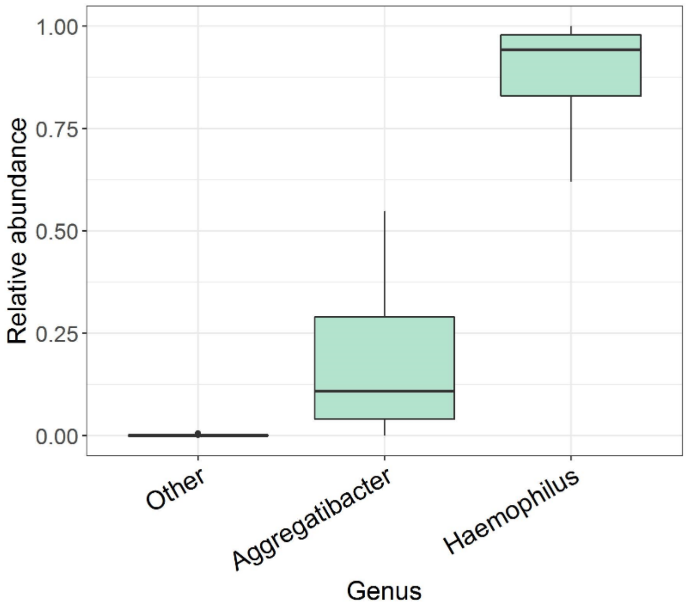

Supplementary Figure 3 Relative abundances of genera in family *Pasteurellaceae* of all study participants. Due to very low relative abundances of genera other than *Haemophilus* and *Aggregatibacter*, other genera (*Actinobacillus*, *Pasteurella*, *Mannheimia*, *Gallibacterium*, *Avibacterium*, *Basfia*, *Chelonobacter*, *Necropsobacter*, *Otariodibacter*, *Vespertiliibacter*, *Frederiksenia*, and *Mesocricetibacter*) in family Pasteurellaceae, were merged together for analysis. **(a)** Bar plots of the relative abundances in each saliva sample. *Haemophilus* accounted for at least half of the abundances in Pasteurellaceae in each saliva sample. **(b)** Box plot of summarized results. *Haemophilus* was the most abundant genus of *Pasteurellaceae*, followed by *Aggregatibacter*. Kruskal-Wallis test: $p < 0.001$. Post-hoc analysis, $p < 0.05$, between any of the two groups. Box: Q1, median, and Q3.

a

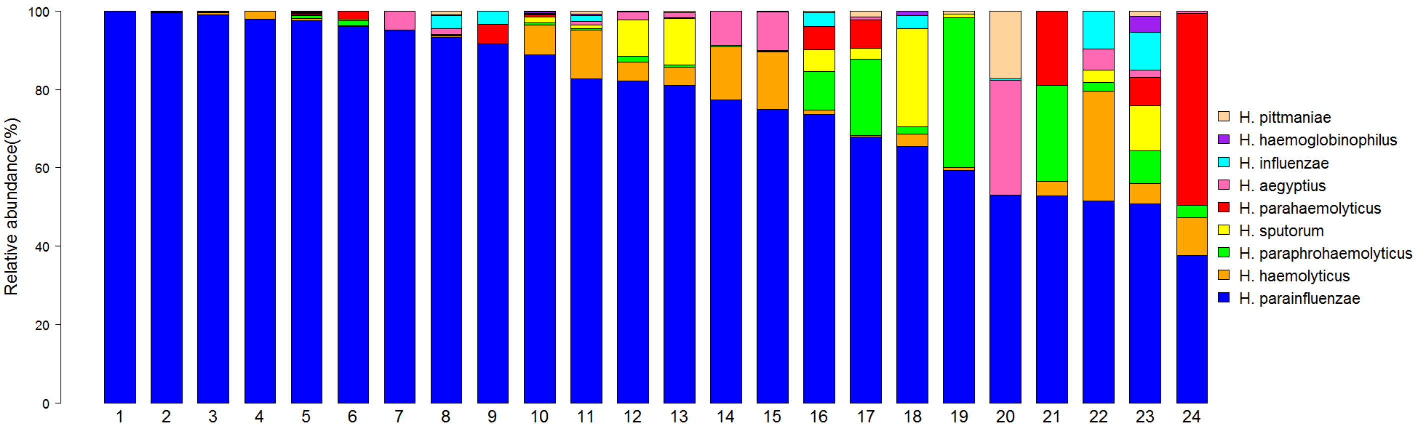

b

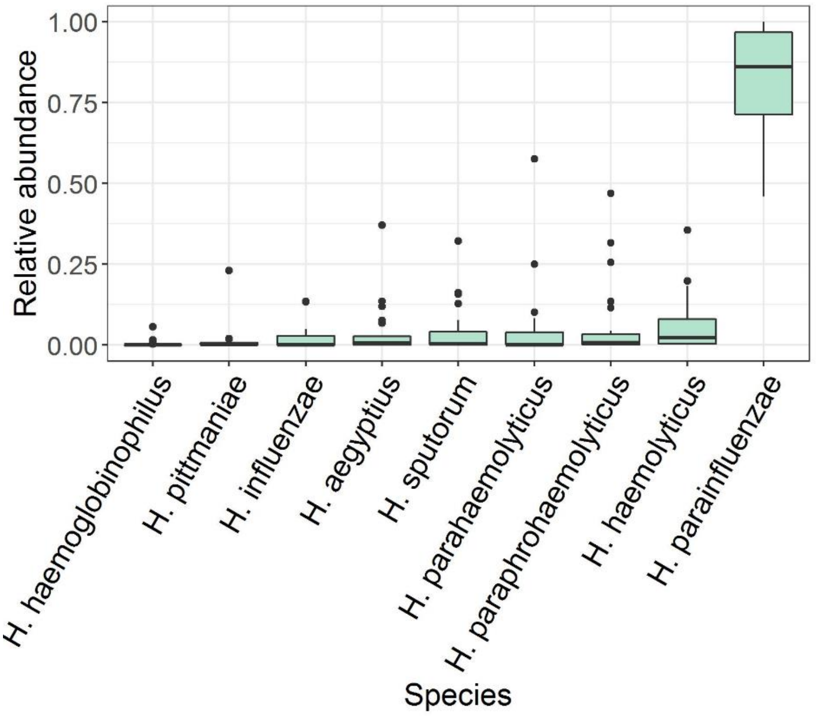

Supplementary Figure 4 Relative abundances of species in *Haemophilus*. (a) Bar plot of relative abundances of each saliva sample from all study participants. Relative abundance of *H. parainfluenzae* was the highest in most of the saliva samples. **(b)** Box plot of summarized results. *H. parainfluenzae* was the most abundant species in *Haemophilus*. Kruskal-Wallis test: $p < 0.001$. Post-hoc analysis, $p < 0.05$, between *H. parainfluenzae* and any other species, between *H. haemolyticus* and *H. haemoglobinophilus*, and between *H. paraphrohaemolyticus* and *H. haemoglobinophilus*. Box: Q1, median, and Q3; point: outlier.

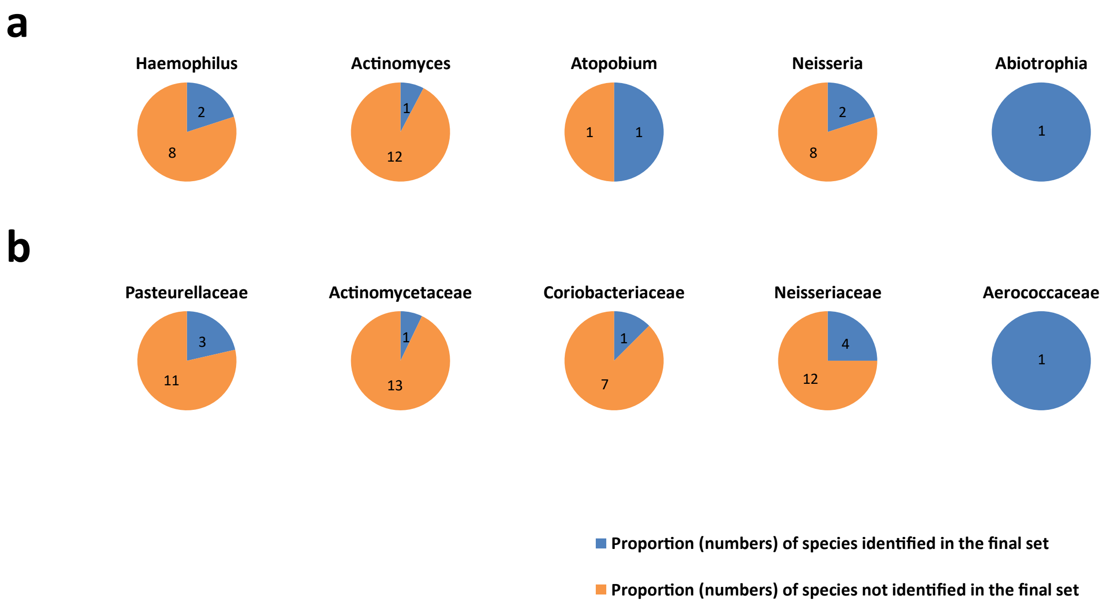

Supplementary Figure 5 Pie chart representing the number of species in specific genus and family containing differentially abundant species with LDA scores greater than 3. (a) *Haemophilus parainfluenzae*, *Actinomyces odontolyticus*, *Atopobium parvulum*, *Neisseria elongate*, and *Abiotrophia defectiva* belong to families. **(b)** Pasteurellaceae, Actinomycetaceae, Coriobacteriaceae, Neisseriaceae, and Aerococcaceae, respectively. The portions of species identified in the final set in specific genus and family are colored blue, while those not identified are in orange. A species in the corresponding genus or family was not counted if the relative abundance was less than 0.01% in every saliva sample. *Abiotrophia defectiva* was the only species counted in *Abiotrophia* and family Aerococcaceae.

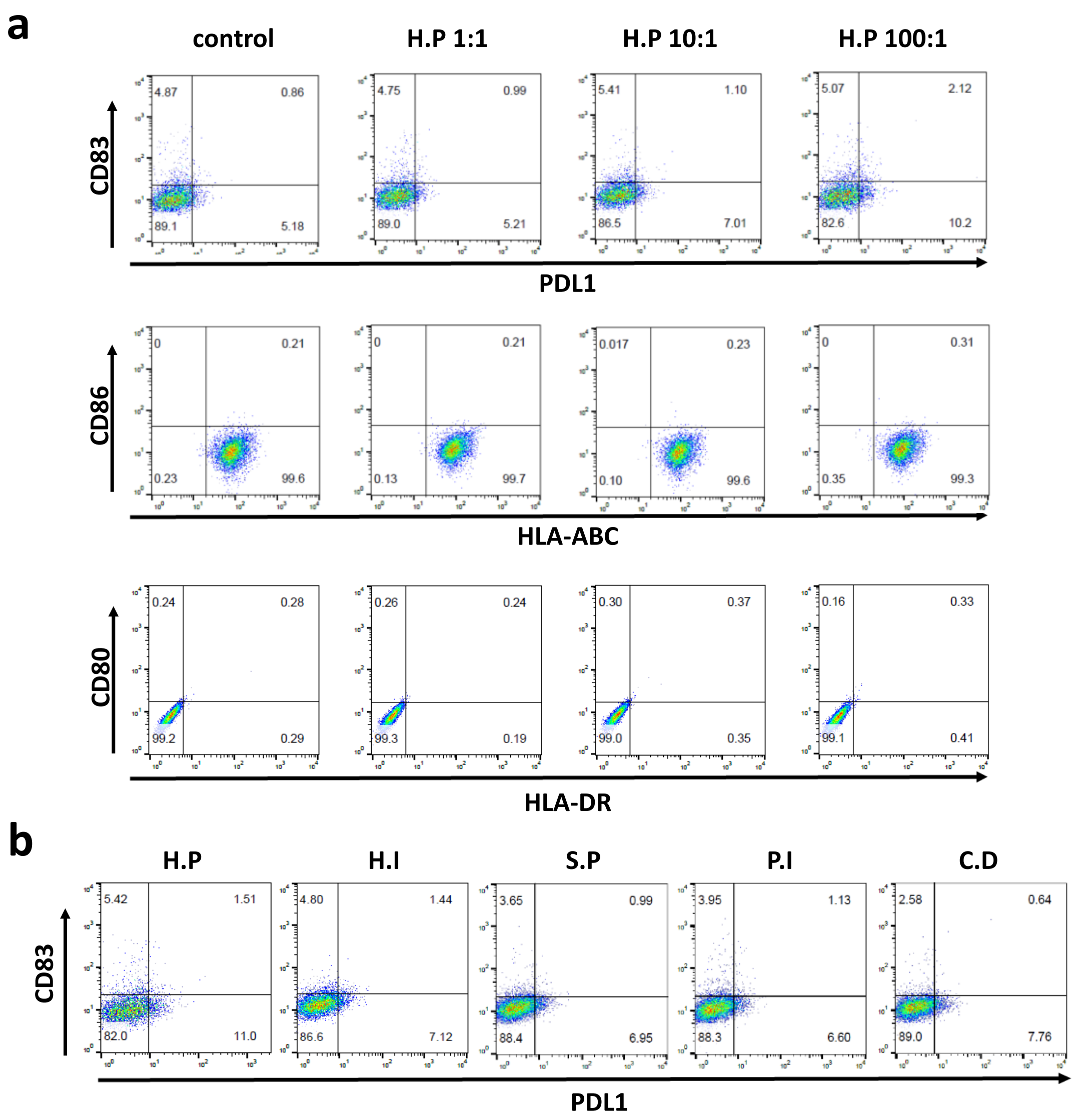

Supplementary Figure 6 Representative dot-plots of surface markers following bacterial treatment. Surface markers of A253 cells were analyzed by flow cytometry. **(a)** Expression of PD-L1, CD83, CD80, CD86, HLA-ABC, and HLA-DR following treatment with *Haemophilus parainfluenzae* at various bacteria-to-cell ratios. **(b)** PD-L1 expression following treatment with various bacteria at a bacteria-to-cell ratio of 100:1. H.P: *Haemophilus parainfluenzae*, H.I: *Haemophilus influenzae*, S.P: *Streptococcus pyogenes*, P.I: *Prevotella intermedia*, C.D: *Clostridium difficile*.

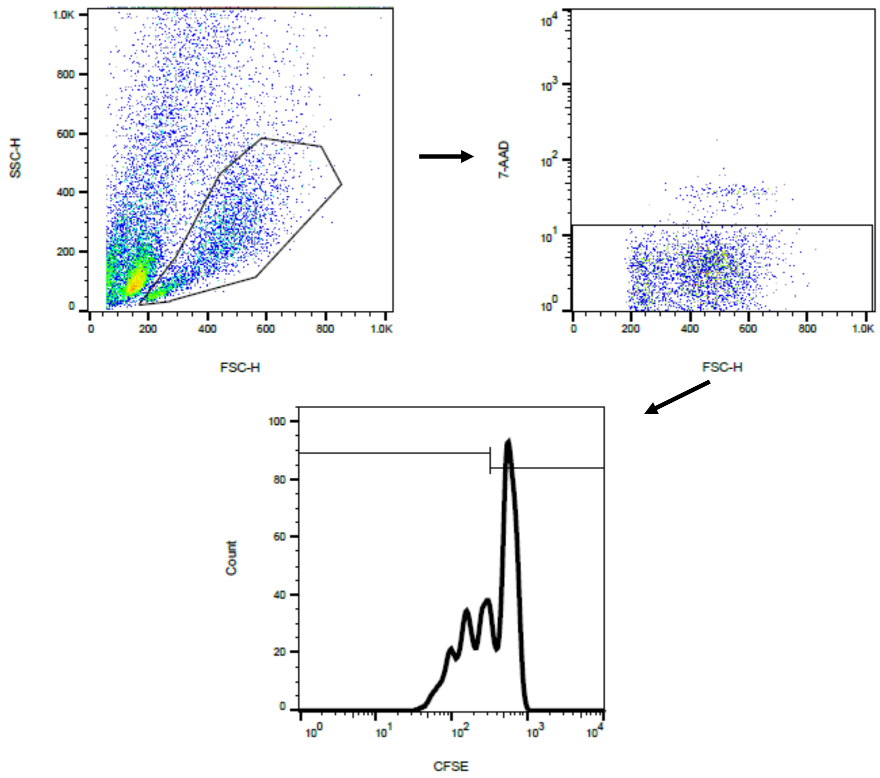

Supplementary Figure 7 Gating strategy for CD4 T cell proliferation.

After co-culture of CD4 T cells with *Haemophilus parainfluenzae*-pretreated A253 cells, FSC-SSC profile was used to distinguish CD4 T cells. Viable cells were identified by 7-AAD negativity. Only the gated cells were analyzed for CFSE intensity.

Supplementary Table 1. Taxa identified in the final set

Taxon	Taxonomic rank	Phylum	Class	Order	Family	LDA score	Direction	*p* value
Gammaproteobacteria	Class	Proteobacteria	-	-	-	4.11	Healthy controls	0.010
Deltaproteobacteria	Class	Proteobacteria	-	-	-	2.21	Healthy controls	0.020
Burkholderiales	Order	Proteobacteria	Betaproteobacteria	-	-	4.11	Healthy controls	0.032
Pasteurellales	Order	Proteobacteria	Gammaproteobacteria	-	-	4.04	Healthy controls	0.014
Cardiobacteriales	Order	Proteobacteria	Gammaproteobacteria	-	-	2.59	Healthy controls	0.012
Desulfovibrionales	Order	Proteobacteria	Deltaproteobacteria	-	-	2.21	Healthy controls	0.020
Pasteurellaceae	Family	Proteobacteria	Gammaproteobacteria	Pasteurellales	-	4.04	Healthy controls	0.014
Aerococcaceae	Family	Firmicutes	Bacilli	Lactobacillales	-	3.07	Healthy controls	0.030
Cardiobacteriaceae	Family	Proteobacteria	Gammaproteobacteria	Cardiobacteriales	-	2.59	Healthy controls	0.012
Haemophilus	Genus	Proteobacteria	Gammaproteobacteria	Pasteurellales	Pasteurellaceae	3.97	Healthy controls	0.012
Aggregatibacter	Genus	Proteobacteria	Gammaproteobacteria	Pasteurellales	Pasteurellaceae	3.21	Healthy controls	0.007
Abiotrophia	Genus	Firmicutes	Bacilli	Lactobacillales	Aerococcaceae	3.07	Healthy controls	0.030
Cardiobacterium	Genus	Proteobacteria	Gammaproteobacteria	Cardiobacteriales	Cardiobacteriaceae	2.59	Healthy controls	0.012
Megasphaera	Genus	Firmicutes	Clostridia	Clostridiales	Veillonellaceae	2.57	pSS patients	0.050
Johnsonella	Genus	Firmicutes	Clostridia	Clostridiales	Lachnospiraceae	2.26	Healthy controls	0.033
Bifidobacterium	Genus	Actinobacteria	Actinobacteria	Bifidobacteriales	Bifidobacteriaceae	2.12	Healthy controls	0.026
Haemophilus parainfluenzae	Species	Proteobacteria	Gammaproteobacteria	Pasteurellales	Pasteurellaceae	3.79	Healthy controls	0.017
Actinomyces odontolyticus	Species	Actinobacteria	Actinobacteria	Actinomycetales	Actinomycetaceae	3.76	pSS patients	0.032
Atopobium parvulum	Species	Actinobacteria	Coriobacteriia	Coriobacteriales	Coriobacteriaceae	3.47	pSS patients	0.032
Neisseria elongata	Species	Proteobacteria	Betaproteobacteria	Neisseriales	Neisseriaceae	3.16	Healthy controls	0.004
Abiotrophia defectiva	Species	Firmicutes	Bacilli	Lactobacillales	Aerococcaceae	3.08	Healthy controls	0.030
Aggregatibacter aphrophilus	Species	Proteobacteria	Gammaproteobacteria	Pasteurellales	Pasteurellaceae	2.99	Healthy controls	0.007
Capnocytophaga granulosa	Species	Bacteroidetes	Flavobacteria	Flavobacteriales	Flavobacteriaceae	2.86	Healthy controls	0.043
Corynebacterium argentoratense	Species	Actinobacteria	Actinobacteria	Actinomycetales	Corynebacteriaceae	2.82	pSS patients	0.041

Granulicatella elegans	Species	Firmicutes	Bacilli	Lactobacillales	Carnobacteriaceae	2.68	Healthy controls	0.024
Leptotrichia shahii	Species	Fusobacteria	Fusobacteria	Fusobacteriales	Leptotrichiaceae	2.63	Healthy controls	0.043
Megasphaera micronuciformis	Species	Firmicutes	Clostridia	Clostridiales	Veillonellaceae	2.60	pSS patients	0.037
Cardiobacterium hominis	Species	Proteobacteria	Gammaproteobacteria	Cardiobacteriales	Cardiobacteriaceae	2.57	Healthy controls	0.008
Pseudomonas geniculata	Species	Proteobacteria	Gammaproteobacteria	Pseudomonadales	Pseudomonadaceae	2.47	pSS patients	0.041
Leptotrichia goodfellowii	Species	Fusobacteria	Fusobacteria	Fusobacteriales	Leptotrichiaceae	2.36	Healthy controls	0.022
Campylobacter gracilis	Species	Proteobacteria	Epsilonproteobacteria	Campylobacterales	Campylobacteraceae	2.35	Healthy controls	0.010
Johnsonella ignava	Species	Firmicutes	Clostridia	Clostridiales	Lachnospiraceae	2.26	Healthy controls	0.033
Kingella denitrificans	Species	Proteobacteria	Betaproteobacteria	Neisseriales	Neisseriaceae	2.23	Healthy controls	0.031
Haemophilus pittmaniae	Species	Proteobacteria	Gammaproteobacteria	Pasteurellales	Pasteurellaceae	2.18	Healthy controls	0.005
Rothia amarae	Species	Actinobacteria	Actinobacteria	Actinomycetales	Micrococcaceae	2.14	pSS patients	0.012
Kingella potus	Species	Proteobacteria	Betaproteobacteria	Neisseriales	Neisseriaceae	2.10	Healthy controls	0.020
Neisseria bacilliformis	Species	Proteobacteria	Betaproteobacteria	Neisseriales	Neisseriaceae	2.07	Healthy controls	0.020
Leptotrichia trevisanii	Species	Fusobacteria	Fusobacteria	Fusobacteriales	Leptotrichiaceae	2.06	Healthy controls	0.040
Prevotella saccharolytica	Species	Bacteroidetes	Bacteroidia	Bacteroidales	Prevotellaceae	2.02	Healthy controls	0.042

pSS: primary Sjögren’s syndrome

Supplementary Table 2. Baseline characteristics of patients in the extension study.

	pSS patients	Non-pSS sicca patients
Number of cases	10	11
Age (year, IQR)	50.5 (46.9 – 60.1)	49.4 (44.9 – 54.2)
Women (n, %)	10 (100)	11 (100)
Duration of clinically apparent xerostomia (months, IQR)	12 (8 – 21)	9 (6 – 24)
Sialoscintigraphy grades		
< grade II (n, %)	8 (80)	9 (82)
= grade II (n, %)	2 (20)	2 (18)
> grade II (n, %)	0 (0)	0 (0)
Positivity of anti-Ro or anti-La (n, %)	4 (40)	0 (0)
Positivity of anti-Ro (n, %)	4 (40)	0 (0)
Positivity of anti-La (n, %)	1 (10)	0 (0)
LSG biopsy focus score ≥1 (no, %)	6 (60)	0 (0)

IQR: interquartile range; pSS: primary Sjögren's syndrome; LSG: labial salivary gland

Supplementary Table 3. Differential abundant taxa between pSS and non-pSS sicca patients

Taxon[1]	Taxonomic rank	Phylum	Class	Order	Family	LDA score	Direction	*p* value
Clostridia	Class	Firmicutes	Clostridia	-	-	4.45	non-pSS sicca	0.029
Gammaproteobacteria	Class	Proteobacteria	Gammaproteobacteria	-	-	4.13	non-pSS sicca	0.011
Clostridiales	Order	Firmicutes	Clostridia	Clostridiales	-	4.45	non-pSS sicca	0.029
Pasteurellales	Order	Proteobacteria	Gammaproteobacteria	Pasteurellales	-	4.12	non-pSS sicca	0.009
Pasteurellaceae	Family	Proteobacteria	Gammaproteobacteria	Pasteurellales	Pasteurellaceae	4.12	non-pSS sicca	0.009
Haemophilus	Genus	Proteobacteria	Gammaproteobacteria	Pasteurellales	Pasteurellaceae	4.11	non-pSS sicca	0.007
Megasphaera	Genus	Firmicutes	Clostridia	Clostridiales	Veillonellaceae	3.09	non-pSS sicca	0.029
Kingella	Genus	Proteobacteria	Betaproteobacteria	Neisseriales	Neisseriaceae	2.65	non-pSS sicca	0.041
Dialister	Genus	Firmicutes	Clostridia	Clostridiales	Veillonellaceae	2.29	non-pSS sicca	0.035
Anaeroglobus	Genus	Firmicutes	Clostridia	Clostridiales	Veillonellaceae	2.12	non-pSS sicca	0.027
Neisseria subflava	Species	Proteobacteria	Betaproteobacteria	Neisseriales	Neisseriaceae	4.07	pSS	0.035
Haemophilus parainfluenzae	Species	Proteobacteria	Gammaproteobacteria	Pasteurellales	Pasteurellaceae	4.00	non-pSS sicca	0.009
Campylobacter concisus	Species	Proteobacteria	Epsilonproteobacteria	Campylobacterales	Campylobacteraceae	3.83	non-pSS sicca	0.049
Veillonella dispar	Species	Firmicutes	Clostridia	Clostridiales	Veillonellaceae	3.72	non-pSS sicca	0.049
Campylobacter showae	Species	Proteobacteria	Epsilonproteobacteria	Campylobacterales	Campylobacteraceae	3.29	non-pSS sicca	0.049
Megasphaera micronuciformis	Species	Firmicutes	Clostridia	Clostridiales	Veillonellaceae	3.09	non-pSS sicca	0.029
Prevotella pallens	Species	Bacteroidetes	Bacteroidia	Bacteroidales	Prevotellaceae	3.00	non-pSS sicca	0.024
Mycoplasma salivarium	Species	Tenericutes	Mollicutes	Mycoplasmatales	Mycoplasmataceae	2.96	non-pSS sicca	0.008
Kingella oralis	Species	Proteobacteria	Betaproteobacteria	Neisseriales	Neisseriaceae	2.61	non-pSS sicca	0.012
Alloprevotella rava	Species	Bacteroidetes	Bacteroidia	Bacteroidales	Prevotellaceae	2.44	non-pSS sicca	0.034
Mogibacterium neglectum	Species	Firmicutes	Clostridia	Clostridiales	Mogibacteriaceae	2.32	non-pSS sicca	0.045
Campylobacter gracilis	Species	Proteobacteria	Epsilonproteobacteria	Campylobacterales	Campylobacteraceae	2.32	non-pSS sicca	0.035
Dialister pneumosintes	Species	Firmicutes	Clostridia	Clostridiales	Veillonellaceae	2.26	non-pSS sicca	0.031
Anaeroglobus geminatus	Species	Firmicutes	Clostridia	Clostridiales	Veillonellaceae	2.09	non-pSS sicca	0.027

pSS: primary Sjögren's syndrome

[1] Differential abundant taxa not meeting the filtering criteria were not presented.

Supplementary References

61 Kilian, M. & Schiott, C. R. Haemophili and related bacteria in the human oral cavity. *Arch. Oral. Biol.* **20**, 791-IN797 (1975).

62 Liljemark, W. F. *et al.* Distribution of oral Haemophilus species in dental plaque from a large adult population. *Infect. Immun.* **46**, 778-786 (1984).

63 Correa, J. D. *et al.* Subgingival microbiota dysbiosis in systemic lupus erythematosus: association with periodontal status. *Microbiome* **5**, 34 (2017).

64 Tian, N. *et al.* Salivary Gluten Degradation and Oral Microbial Profiles in Healthy Individuals and Celiac Disease Patients. *Appl. Environ. Microbiol.* **83** (2017).

65 Bruserud, O. *et al.* Oral microbiota in autoimmune polyendocrine syndrome type 1. *J. Oral Microbiol.* **10**, 1442986 (2018).

66 Iwasawa, K. *et al.* Dysbiosis of the salivary microbiota in pediatric-onset primary sclerosing cholangitis and its potential as a biomarker. *Sci. Rep.* **8**, 5480 (2018).

67 Said, H. S. *et al.* Dysbiosis of salivary microbiota in inflammatory bowel disease and its association with oral immunological biomarkers. *DNA Res.* **21**, 15-25 (2014).

68 Goel, R. M. *et al.* Streptococcus Salivarius: A Potential Salivary Biomarker for Orofacial Granulomatosis and Crohn's Disease? *Inflamm. Bowel Dis.* **25** (2019).

69 Nakagome, S. *et al.* Confounding effects of microbiome on the susceptibility of TNFSF15 to Crohn's disease in the Ryukyu Islands. *Hum. Genet.* **136**, 387-397 (2017).

70 Xun, Z., Zhang, Q., Xu, T., Chen, N. & Chen, F. Dysbiosis and Ecotypes of the Salivary Microbiome Associated With Inflammatory Bowel Diseases and the Assistance in Diagnosis of Diseases Using Oral Bacterial Profiles. *Front. Microbiol.* **9**, 1136 (2018).

71 Kouno, M. *et al.* Dysbiosis of oral microbiota in palmoplantar pustulosis patients. *J. Dermatol. Sci.* **93**, 67-69 (2019).

72 Batty, I. Actinomyces odontolyticus, a new species of actinomycete regularly isolated from deep carious dentine. *J. Pathol. Bacteriol.* **75**, 455-459 (1958).

73 Belstrom, D. *et al.* Salivary microbiota and inflammation-related proteins in patients with psoriasis. *Oral Dis.* **26** 677-687 (2020).

74 de Groot, P. F. *et al.* Distinct fecal and oral microbiota composition in human type 1 diabetes, an observational study. *PloS one* **12**, e0188475 (2017).

75 Cao, Y. *et al.* Comparative Analyses of Subgingival Microbiome in Chronic Periodontitis Patients with and Without IgA Nephropathy by High Throughput 16S rRNA Sequencing. *Cell. Physiol. Biochem.* **47**, 774-783 (2018).

76 CATO, E. P. Transfer of Peptostreptococcus parvulus (Weinberg, Nativelle, and PrÉvot 1937) Smith 1957 to the Genus Streptococcus: Streptococcus parvulus (Weinberg, Nativelle, and

PrÉvot 1937) comb, nov., nom. rev., emend. *Int. J. Syst. Evol. Microbiol.* **33**, 82-84 (1983).

77 Collins, M. D. & Wallbanks, S. Comparative sequence analyses of the 16S rRNA genes of Lactobacillus minutus, Lactobacillus rimae and Streptococcus parvulus: Proposal for the creation of a new genus Atopobium. *FEMS Microbiol. Lett.* **95**, 235-240 (1992).

78 Olsen, I., Johnson, J. L., Moore, L. V. & Moore, W. E. Lactobacillus uli sp. nov. and Lactobacillus rimae sp. nov. from the human gingival crevice and emended descriptions of lactobacillus minutus and Streptococcus parvulus. *Int. J. Syst. Bacteriol.* **41**, 261-266 (1991).

79 Riggio, M. P. *et al.* Molecular identification of bacteria on the tongue dorsum of subjects with and without halitosis. *Oral Dis*. **14**, 251-258 (2008).

80 Ye, Z. *et al.* A metagenomic study of the gut microbiome in Behcet's disease. *Microbiome* **6**, 135 (2018).

81 Bovre, K. & Holten, E. Neisseria elongata sp.nov., a rod-shaped member of the genus Neisseria. Re-evaluation of cell shape as a criterion in classification. *J. Gen. Microbiol*. **60**, 67-75 (1970).

82 Piccolo, M. *et al.* Salivary Microbiota Associated with Immunoglobulin A Nephropathy. *Microb. Ecol.* **70**, 557-565 (2015).

83 Bouvet, A., GRIMONT, F. & GRIMONT, P. A. D. Streptococcus defectivus sp. nov. and Streptococcus adjacens sp. nov., Nutritionally Variant Streptococci from Human Clinical Specimens. *Int. J. Sys. Bacteriol.* **39**, 290-294 (1989).

84 Kawamura, Y. *et al.* Transfer of Streptococcus adjacens and Streptococcus defectivus to Abiotrophia gen. nov. as Abiotrophia adiacens comb. nov. and Abiotrophia defectiva comb. nov., respectively. *Int. J. Sys. Bacteriol.* **45**, 798-803 (1995).

85 Kanamoto, T., Eifuku-Koreeda, H. & Inoue, M. Isolation and properties of bacteriolytic enzyme-producing cocci from the human mouth. *FEMS Microbiol. Lett.* **144**, 135-140 (1996).
