# Supplementary material for: Salivary dysbiosis in Sjögren’s syndrome and a commensal-mediated immunomodulatory effect of salivary gland epithelial cells
Source: NPJ Biofilms Microbiomes. 2021 Mar 11;7:21. doi: 10.1038/s41522-021-00192-w (PMC7952914; doi:10.1038/s41522-021-00192-w)
Supplement: Supplementary file 2 — Reporting Summary [file 41522_2021_192_MOESM2_ESM.pdf]

nature research

Corresponding author(s): Prof. Song-chou Hsieh, MD, PhD
Prof. Shu-fen Wu, PhD
Prof. Chin Li, PhD

Last updated by author(s): Oct 22, 2020

Reporting Summary

Nature Research wishes to improve the reproducibility of the work that we publish. This form provides structure for consistency and transparency in reporting. For further information on Nature Research policies, see our Editorial Policies and the Editorial Policy Checklist.

Statistics

For all statistical analyses, confirm that the following items are present in the figure legend, table legend, main text, or Methods section.

n/a	Confirmed	
☐	☒	The exact sample size (*n*) for each experimental group/condition, given as a discrete number and unit of measurement
☐	☒	A statement on whether measurements were taken from distinct samples or whether the same sample was measured repeatedly
☐	☒	The statistical test(s) used AND whether they are one- or two-sided<br>*Only common tests should be described solely by name; describe more complex techniques in the Methods section.*
☒	☐	A description of all covariates tested
☐	☒	A description of any assumptions or corrections, such as tests of normality and adjustment for multiple comparisons
☐	☒	A full description of the statistical parameters including central tendency (e.g. means) or other basic estimates (e.g. regression coefficient) AND variation (e.g. standard deviation) or associated estimates of uncertainty (e.g. confidence intervals)
☒	☐	For null hypothesis testing, the test statistic (e.g. *F*, *t*, *r*) with confidence intervals, effect sizes, degrees of freedom and *P* value noted<br>*Give P values as exact values whenever suitable.*
☒	☐	For Bayesian analysis, information on the choice of priors and Markov chain Monte Carlo settings
☒	☐	For hierarchical and complex designs, identification of the appropriate level for tests and full reporting of outcomes
☐	☒	Estimates of effect sizes (e.g. Cohen's *d*, Pearson's *r*), indicating how they were calculated

Our web collection on statistics for biologists contains articles on many of the points above.

Software and code

Policy information about availability of computer code

Data collection	Participants data were collected with Microsoft Excel 2016. Flowcytometry data were processed using FlowJo™. Sequencing data of 16S ribosomal DNA were processed using CLC Genomic Workbench v.8.5.
Data analysis	Statistical analyses were performed in SPSS for Windows v.21.0. Microbiota data were analyzed by LEfSe (http://huttenhower.sph.harvard.edu/galaxy/).

For manuscripts utilizing custom algorithms or software that are central to the research but not yet described in published literature, software must be made available to editors and reviewers. We strongly encourage code deposition in a community repository (e.g. GitHub). See the Nature Research guidelines for submitting code & software for further information.

Data

Policy information about availability of data

All manuscripts must include a data availability statement. This statement should provide the following information, where applicable:
- Accession codes, unique identifiers, or web links for publicly available datasets
- A list of figures that have associated raw data
- A description of any restrictions on data availability

The data that support the findings of this study are available from the corresponding author, upon reasonable request.

Field-specific reporting

Please select the one below that is the best fit for your research. If you are not sure, read the appropriate sections before making your selection.

☒ Life sciences ☐ Behavioural & social sciences ☐ Ecological, evolutionary & environmental sciences

For a reference copy of the document with all sections, see nature.com/documents/nr-reporting-summary-flat.pdf

Life sciences study design

All studies must disclose on these points even when the disclosure is negative.

Sample size	Sample size determined according to past study results
Data exclusions	Only data from eligible participants were included in the analysis. The criteria were pre-established.
Replication	All experiments were repeated for at least three times.
Randomization	NA
Blinding	NA

Reporting for specific materials, systems and methods

We require information from authors about some types of materials, experimental systems and methods used in many studies. Here, indicate whether each material, system or method listed is relevant to your study. If you are not sure if a list item applies to your research, read the appropriate section before selecting a response.

Materials & experimental systems

n/a	Involved in the study	
☐	☒	Antibodies
☐	☒	Eukaryotic cell lines
☒	☐	Palaeontology and archaeology
☒	☐	Animals and other organisms
☐	☒	Human research participants
☒	☐	Clinical data
☒	☐	Dual use research of concern

Methods

n/a	Involved in the study	
☒	☐	ChIP-seq
☐	☒	Flow cytometry
☒	☐	MRI-based neuroimaging

Antibodies

Antibodies used	anti-human PD-L1 blocking antibody (clone: 29E.2A3, Biolegend)fluorescent antibodies against PD-L1-allophycocyanin (APC, clone: MIH1), CD80-phycoerythrin (PE, clone: L307) and CD86-PE (clone: IT2.2) were purchased from BD Bioscience. The fluorescent antibodies against CD83-PE (clone: HB15), HLA-ABC-Alexa Fluor 488 (clone: W6/32), and HLA-DR- Alexa Fluor 488 (clone: L243) were purchased from Biolegend.
Validation	Validation was supported by the suppliers including Biolegend and BD Bioscience.

Eukaryotic cell lines

Policy information about cell lines

Cell line source(s)	A-253 (BCRC 60367 = ATCC® HTB-41™) : human submaxillary gland
Authentication	Authentication provided by Biosource Collection and Research Center, Taiwan
Mycoplasma contamination	not tested
Commonly misidentified lines (See ICLAC register)	NA

Human research participants

Policy information about studies involving human research participants

Population characteristics	Primary Sjogren's syndrome (pSS) patients; healthy controls; non-pSS sicca patients
Recruitment	Patients: from rheumatology clinic<br>Healthy controls: from community<br>Potential bias: non-pSS sicca patients need to be eligible for a negative labial salivary gland biopsy. It is expected that patients receiving this invasive procedure may be a specific sub-population rendering selection bias.
Ethics oversight	the Research Ethical Committee of Ditmanson Medical Foundation Chia-Yi Christian Hospital (IRB106031),

Note that full information on the approval of the study protocol must also be provided in the manuscript.

Flow Cytometry

Plots

Confirm that:

☒ The axis labels state the marker and fluorochrome used (e.g. CD4-FITC).

☒ The axis scales are clearly visible. Include numbers along axes only for bottom left plot of group (a 'group' is an analysis of identical markers).

☒ All plots are contour plots with outliers or pseudocolor plots.

☒ A numerical value for number of cells or percentage (with statistics) is provided.

Methodology

Sample preparation	CD4 T cell isolation was isolated by negative selection using BD IMag™ Human CD4 T Lymphocyte Enrichment Set - DM.
Instrument	FACSCalibur (BD Bioscience) flow cytometry
Software	FlowJo software v10
Cell population abundance	Isolated CD4 T cell population purity was more than 96%.
Gating strategy	lymphocyte population gated by FSC/SSC<br>7-AAD gating by unstained control

☒ Tick this box to confirm that a figure exemplifying the gating strategy is provided in the Supplementary Information.
